# Machine learning for orbit steering in the presence of nonlinearities

**DOI:** 10.1107/S1600577525002334

**Published:** 2025-04-11

**Authors:** Simona Bettoni, Jonas Kallestrup, Güney Erin Tekin, Michael Böge, Romana Boiger

**Affiliations:** a Paul Scherrer Institute Center for Accelerator Science and Engineering 5232Villigen Switzerland; Utrecht University, The Netherlands

**Keywords:** beam stabilization, machine learning, orbit correction, accelerator stabilization

## Abstract

Circular particle accelerators require precise beam orbit correction to maintain the beam’s trajectory close to the ideal ‘golden orbit’, which is centered within all magnetic elements of the ring, except for slight deviations due to installed experiments. Traditionally, this correction is achieved using methodologies based on the response matrix (RM). The RM elements remain constant when the accelerator’s lattice includes solely linear elements or when a linear approximation is valid for small perturbations, allowing for the calculation of corrector strengths to steer the beam. However, most circular accelerators contain nonlinear magnets, leading to variations in RM elements when the beam experiences large perturbations, rendering traditional methods less effective and necessitating multiple iterations for convergence. To address these challenges, a machine learning (ML)-based approach is explored for beam orbit correction. This approach, applied to synchrotron SLS 2.0 under construction at the Paul Scherrer Institut, is evaluated against and in combination with the standard RM-based method under various conditions. A possible limitation of ML for this application is the potential change in the dimensionality of the ML model after optimization, which could affect performance. A solution to this issue is proposed, improving the robustness and appeal of the ML-based method for efficient beam orbit steering.

## Abstract 2

A machine learning (ML)-based method for beam orbit correction in circular particle accelerators is explored. Traditional correction techniques rely on the response matrix. Nonlinear elements, like those in the upcoming SLS 2.0 at the Paul Scherrer Institut, challenge the effectiveness of response-matrix-based methods. The ML approach promises to handle nonlinearities and large perturbations more efficiently. A potential limitation of ML models—dimensional changes after optimization—is also addressed, and a solution to enhance model robustness is proposed, paving the way for more reliable and efficient beam steering.

## 1. Introduction

Beam orbit correction is a critical aspect for a wide range of accelerators, from those for high-energy physics experiments to synchrotron light sources for biology and chemistry applications. Several methods have been established to steer the beam, with the most commonly used approaches based on response matrix (RM) inversion or singular value decomposition, depending on the dimensions of the beam position monitors (BPM) and correctors. In all these approaches, the terms of the RM are considered constant, an assumption strictly valid only when the machine’s lattice contains solely linear elements.

However, synchrotrons which are equipped with sextupoles and octupoles do not fulfill this condition, especially in cases of large orbit excursions. In these machines, the beam orbit is typically successfully corrected by weakening the error related to these nonlinear effects by means of correction gains smaller than one and iteration of the correction process. As the beam gradually approaches the on-axis condition through the nonlinear magnets, the error becomes more and more negligible, and the orbit can be more efficiently steered.

Given the large number of variables involved (in principle, all possible orbits through the nonlinear magnets and the combinations of corrector strengths), machine learning (ML) is a highly suitable candidate for addressing this type of problem.

ML has garnered significant interest in recent years across numerous fields. In accelerators, its applications can be broadly categorized into two main areas, virtual diagnostics and optimization of machine performance. Maximizing availability is a crucial objective for accelerators, making non-invasive methods to characterize the beam highly desirable. Virtual diagnostics enable the determination of beam properties using a pre-trained model, thereby avoiding interruptions to machine operation (Kaiser *et al.*, 2024 ▸; Hanuka *et al.*, 2021 ▸; Bettoni *et al.*, 2024 ▸).

Another area of ML application in accelerators involves optimizing machine performance, such as maximizing photon intensity in free-electron laser (FEL) facilities (Duris *et al.*, 2020 ▸), or stabilizing the beam in synchrotron light sources by stabilizing the beam transverse size with respect to the variation of the insertion devices’ properties (Leemann *et al.*, 2019 ▸; Hellert *et al.*, 2024 ▸) or the beam orbit (Schirmer, 2019 ▸; Bai *et al.*, 2022 ▸; Li *et al.*, 2023 ▸). These works explored the use of neural networks (NNs) as an improved method for beam orbit steering. In the case of a machine having solely linear magnets the response of the machine to the corrector excitations is linear, and the corrector strengths to correct any distortion from the target orbit are those producing an orbit equal to the difference between the target orbit and the actual one. For accelerators that include nonlinear elements, not only does the response of the machine depend on the actual orbit, but the resulting orbit is also not simply the sum of the orbits measured under different conditions. The works cited above considered the first aspect, and experimental demonstration has shown that this is a valid approach to accelerate orbit correction. In our approach, we also account for the second aspect. Specifically, for a given orbit, we perform a loop of orbit corrections to determine the *optimal* corrector strengths. In other words, in previous work NNs determined the corrector strengths to produce an orbit equal in amplitude and opposite in sign to the difference between the actual and target orbits, assuming that adding this orbit to the actual one would yield the target orbit. Our approach here directly associates the actual orbit with the corrector strengths required to achieve the target orbit. This methodology is expected to address effectively both of the aforementioned aspects, and therefore improve even more the orbit correction in the presence of nonlinearities.

We applied this ML method to SLS 2.0, the synchrotron under construction at the Paul Scherrer Institut (PSI), described in Section 2. Section 3 recalls the standard procedure for beam orbit correction based on the determination of the RM, and its limitations in the presence of nonlinearities in the machine. It is beyond the scope of this article to give an exhaustive description of ML, but in Section 4 we provide an overview of relevant concepts to aid the understanding of the present work. Finally, in Section 5 we present the findings of applying the ML approach to SLS 2.0. We considered different families of perturbations in the lattice, and we used ML as a standalone application and in combination with the standard RM-based approach as well. In Section 6 we introduce a methodology aimed at addressing a limitation of ML approaches, namely the variability of the parameter space after the model has been trained, as this is a potentially important limitation of the use of ML methods for orbit correction. This constitutes a significant advancement in the application of ML techniques for accelerators.

## 2. Swiss Light Source 2.0

The Swiss Light Source (SLS) at the PSI has been a cornerstone of research for both Swiss and international scientific communities for more than two decades. The facility has enabled numerous breakthroughs in various fields of photon science by providing high-quality photon beams to 16 beamlines. However, advances in technology and increasing scientific demand have necessitated an upgrade to maintain its leading position. In September 2023, SLS was decommissioned to make way for SLS 2.0, a state-of-the-art fourth-generation light source. The upgrade to SLS 2.0 primarily focuses on the optimization of the lattice, aiming to reduce the horizontal beam emittance by more than a factor of 40, and thus massively increase the photon beam brightness. Table 1 ▸ outlines the most relevant parameters for the discussion in this article, and provides a comparison with those of SLS.

**Figure 1:**
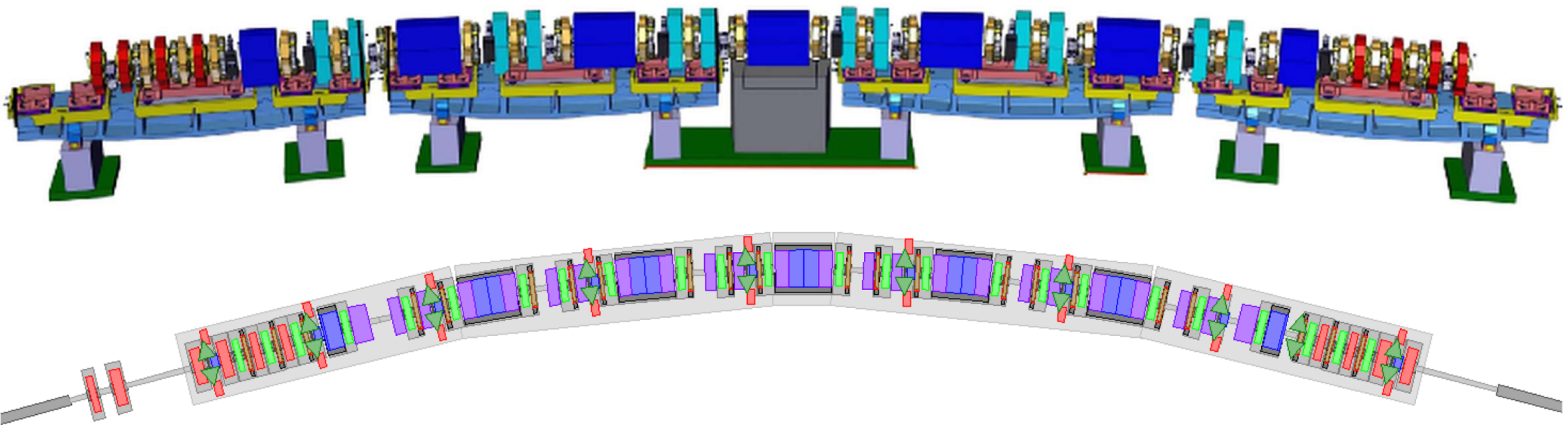
(Top) Girder layout of one out of twelve arcs of SLS 2.0 consisting of four remotely adjustable girders and a central monolithic plinth. Each girder has a length of about 4 m, and the distance between two of them is aboout 3 m. (Bottom) Corresponding schematic magnet arrangement (BPMs are represented by double arrows). Apart from the last downstream BPM, they are accompanied by adjacent pairs of horizontal and vertical correctors.

Fig. 1 ▸ shows the layout of one of the arcs of SLS 2.0. Following the example of SLS it was decided to place pairs of horizontal and vertical correctors adjacent to BPMs in the twelve arcs of SLS 2.0 in order to enable orbit correction by direct inversion of the resulting square RM. Additional BPMs are being installed in the machine to improve dispersion correction (last downstream BPM in the arcs) and to provide redundancy when applying the linear optics from closed orbits (*LOCO*) algorithm (Safranek, 1997 ▸). The maximum kick strength (see Table 1 ▸) has been optimized to guarantee the necessary precision in orbit steering for static bumps and slow and fast orbit correction feedbacks, and the need for correction of machine imperfections, assuming a reasonable set of element misalignments (correlated misalignments from girders and uncorrelated errors from individual magnets) as detailed by Streun *et al.* (2023 ▸).

At the time of this work the installation of SLS 2.0 was underway. For this reason, we performed all our studies using simulation codes: the virtual accelerator first, based on *MAD-X* (https://archive.org/details/manualzilla-id-6906976), and *pyAT* (Rogers *et al.*, 2017 ▸) later.

## 3. Orbit correction in synchrotrons

Orbit correction is a critical aspect of beam stabilization in accelerators and several algorithms have been developed to address this aspect. In the following sections, we will focus on an approach based on the use of orbit excitation induced by changes in the corrector strengths. In particular, we will describe the methodology for machines containing solely linear magnets (dipoles and quadrupoles) in Section 3.1. We will discuss the limitations of this approach when higher-order magnets (such as sextupoles and octupoles) are present in the accelerator’s lattice in Section 3.2.

### 3.1. Linear orbit response

The usual orbit correction method in synchrotrons is based on either a modeled or a measured RM, **R**. This matrix may be divided into four blocks,
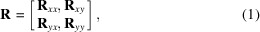
where *x* and *y* are the transverse horizontal and vertical dimension, respectively. The sub-matrix **R**_*xx*_ (**R**_*yy*_) is the *pure* block, since it describes the beam orbit response in the plane of the corrector excitation. The off-diagonal blocks, **R**_*xy*_ and **R**_*yx*_, describe the RM coupling terms, since they contain the orbit response in the plane orthogonal to that where the correctors are varied. Each block has a size of *M* × *N*, where *M* and *N* are the number of the BPMs and correctors, respectively.

In this section we will assume that the coupling has been previously corrected using the skew elements in the machine, implying that the coupling blocks are null. We will focus on the transverse plane *x*, but all the considerations are valid for the vertical plane *y* as well. The (*i*, *j*)th element of each block describes the orbit change at the *i*th BPM due to an excitation induced by the corrector magnet *j*,
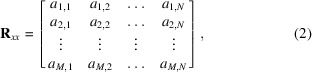
where the *a*_*i*,*j*_ elements are the derivatives of the orbit variation at a specific location along the machine (typically at a BPM) with respect to the strength of the corrector used to induce the orbit oscillation. If the machine contains only dipoles and quadrupoles, without any higher-order multipoles, the terms of the RM are constants and can be calculated analytically as [see, for example, Chao *et al.* (2013 ▸)]
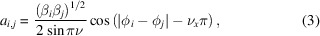
where ν_*x*_ is the horizontal tune, ϕ_*i*_ − ϕ_*j*_ the phase advance between the considered BPM and corrector, and β_*i*_ and β_*j*_ are the Twiss parameters at these locations in the *x* plane.

In linear machines, the change in beam position Δ**x** can be reproduced by a series of corrector setting changes Δ**θ**_*x*_,
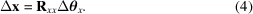
The objective of the beam orbit steering is to determine the Δ**θ** which, when added to the actual corrector strengths, produces the target orbit. For simplicity, and without loss of generality, we assume that the target orbit is that corresponding to the zero orbit. For a square RM, Δ**x** can be obtained by matrix inversion,
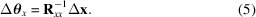
The corrector strengths to steer the beam to the target orbit are determined by subtracting the deflection vector Δ**θ** from the current set point of the correctors. Pseudo-inversion of the RM using *e.g.* singular-value decomposition (SVD) may be considered for non-square RMs. For the inversion, even of square RMs, it is essential that the RM is well conditioned, since small singular values of the RM can funnel noise into the correction; this may be solved using either an eigenvalue cut or Tikhonov regularization (Friedman & Bozoki, 1994 ▸; Tang & Krinsky, 1993 ▸). In the following we consider the RM-based approach based on matrix inversion, which is the approach followed at SLS and that will be used at SLS 2.0.

### 3.2. Effect of nonlinearities

**Figure 2:**
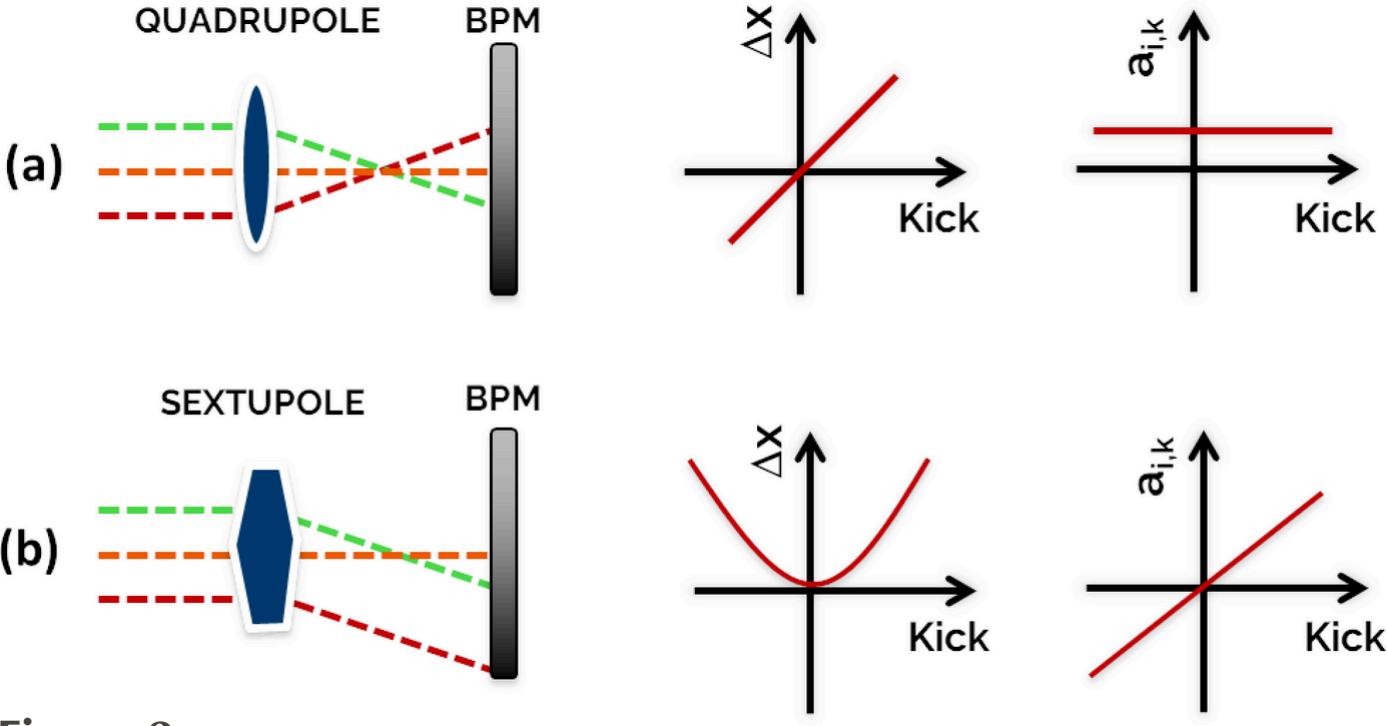
Beam passing through (*a*) a quadrupole and (*b*) a sextupole, assuming different offsets, corresponding variation in the transverse orbit Δ*x* due to the excitation kick by a corrector, and resulting term of the RM.

As outlined above, the RM method inherently assumes linear optics. However, the next generation of light sources, such as SLS 2.0, employ strong nonlinear magnets such as sextupoles and octupoles to correct and control the large chromatic and amplitude-dependent tune shifts. With these magnets, the linearity of the response matrix required for the validity of equations (2)–(5) is no longer guaranteed. As schematically illustrated in Fig. 2 ▸, each element of the RM depends on the beam orbit as the beam passes off-axis through one of the nonlinear magnets along the machine.

As an example, the horizontal pure block will look like
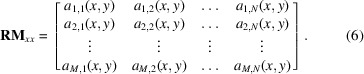
Due to nonlinear coupling arising from high-order magnets (either normal or skew), the *a*_*i*,*j*_ terms of the RM depend on both the horizontal and vertical orbit and, furthermore, they are functions of the beam orbit through the nonlinear magnets.

**Figure 3:**
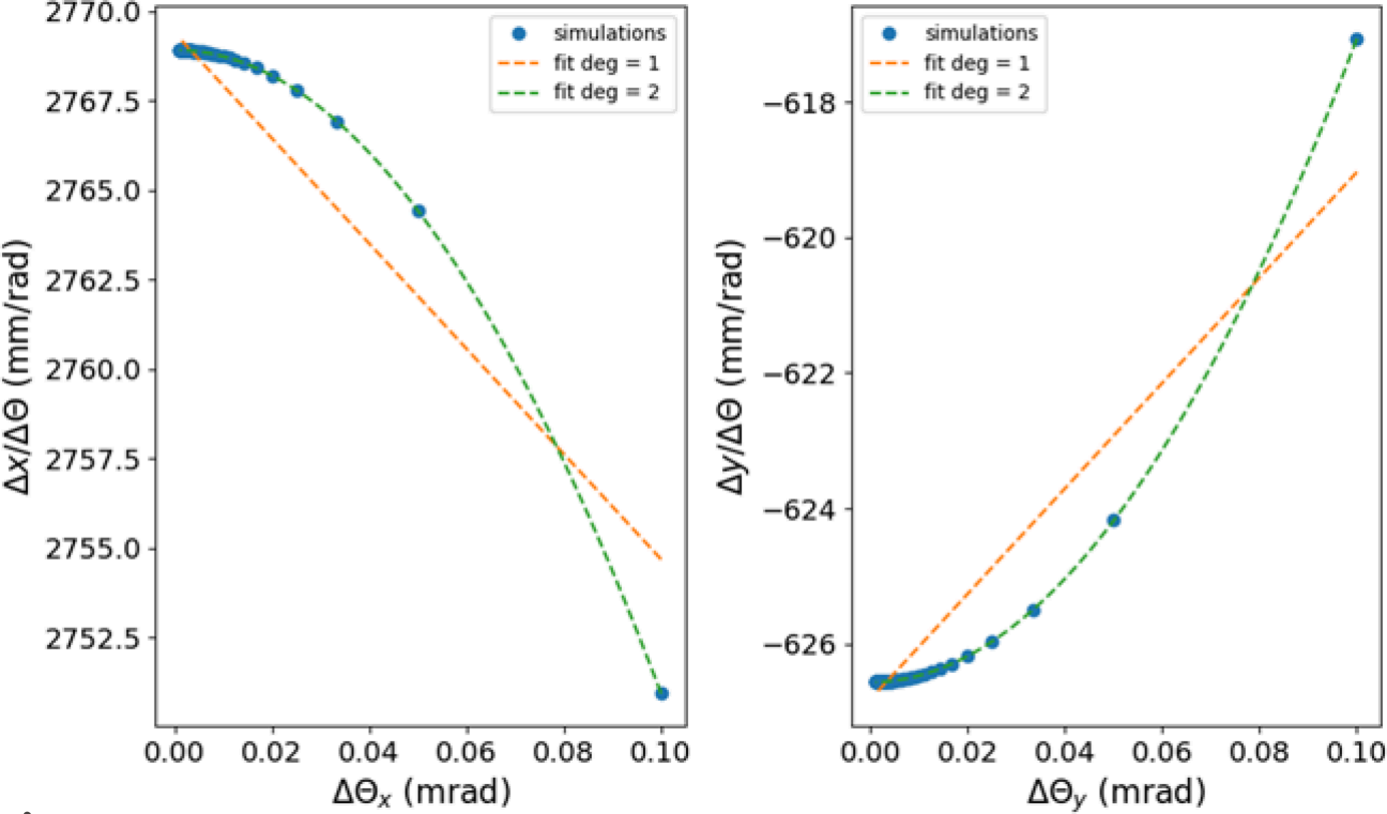
(Left) Horizontal and (right) vertical response terms for a single BPM as a function of the strength of the (left) horizontal and (right) vertical corrector kick. For a machine containing uniquely linear magnets, the terms are expected to be constant. In the case of the SLS 2.0 lattice, where sextupoles and octupoles are present, we observe a dependence of the machine response on the kick amplitude.

We computed the *a*_*i*,*j*_ terms of the SLS 2.0 **RM**_*xx*_ and **RM**_*yy*_ sub-matrices as a function of the corrector excitation using the tracking code *pyAT* to verify the impact of this effect. As shown in Fig. 3 ▸, the response of the beam orbit to a corrector’s excitation is not constant with the strength of the corrector.

**Figure 4:**
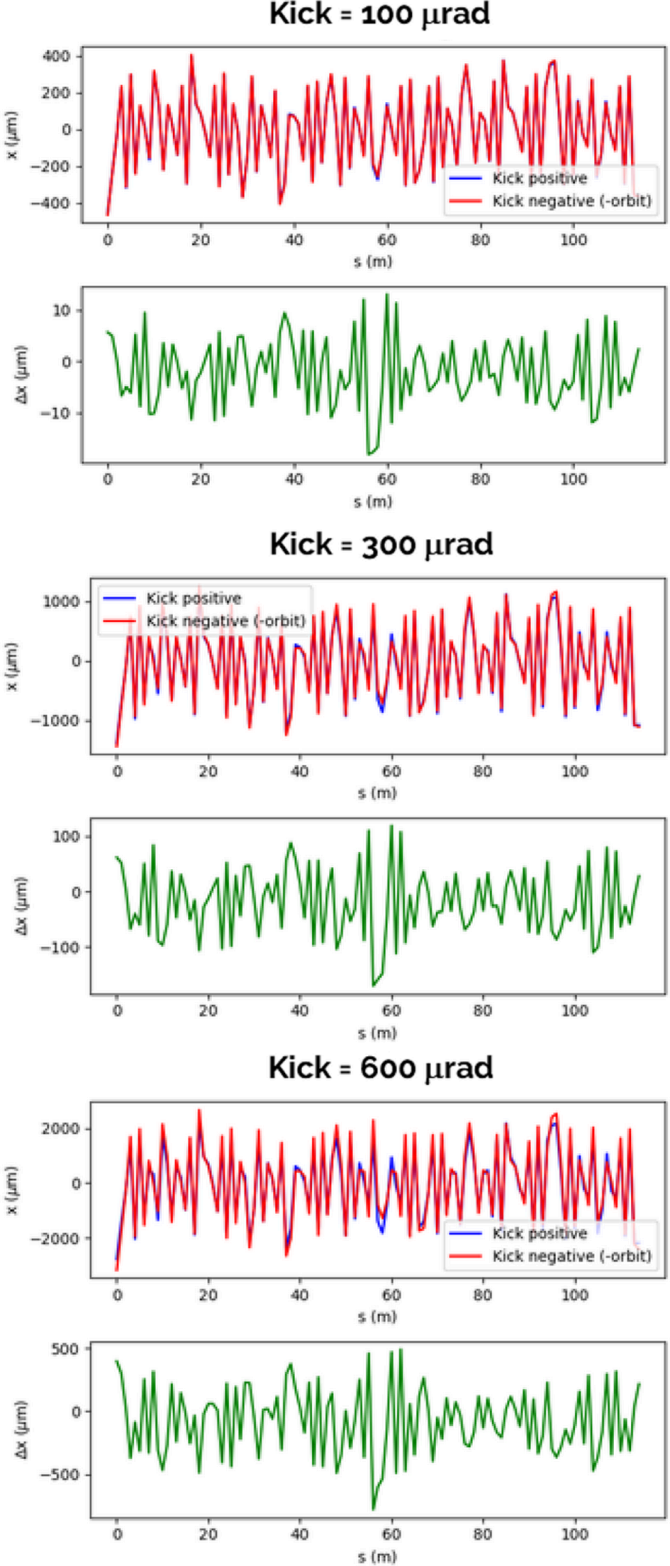
Horizontal orbits at one BPM varying the strength of a corrector assuming an excitation angle of +kick (red line, top plots) and −kick (blue line, top plots) and the difference between the two orbits (green, bottom plots). We repeated the simulations for several kick strengths in the range 100–600 rad, the upper limit being the maximum corrector strength in SLS 2.0. The value of the kick strength is reported above each top plot.

The impact of the nonlinearity of the machine’s response is not a negligible effect, as evident from the plots shown in Fig. 4 ▸. These plots illustrate the orbit responses for both positive and negative kick strengths of equal magnitude and highlight the discrepancies between these two scenarios.

Even for a kick strength equal to one-sixth of the maximum kick strength in the horizontal plane (see Table 1 ▸), we observe a difference of approximately 20 µm between the positive and negative kick orbits. For a corrector strength equal to the maximum strength expected in the horizontal plane, this orbit difference increases up to 1 mm. This makes the machine handling difficult when correcting down to the ‘golden orbit’ from the corresponding very large orbits.

The orbit correction algorithms discussed in Section 3.1 perform effectively even in the presence of nonlinear magnets, albeit requiring multiple iterations to achieve convergence. The terms of the RM can be expanded using a Taylor series,
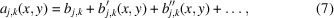
where *b*_*j*,*k*_ is determined by the linear magnets in the machine, whereas 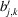 and 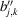 are influenced by the contributions from the higher-order magnets. The difference between *a*_*j*,*k*_ and *b*_*j*,*k*_ decreases as the beam orbit aligns with the higher-order multipole magnets axis due to the iterative orbit correction process.

ML presents a promising approach for beam orbit control, given the large number of parameters involved, *e.g.* any potential beam orbit in both planes and the numerous combinations of corrector strengths. Implementing an ML-based strategy could significantly speed up orbit correction processes, particularly for large orbit excursions. As a demonstration, we applied this methodology to SLS 2.0. However, the approach is equally applicable to any other such machine, high-energy physics machine, light source or any other kind of accelerator, where nonlinearities may affect the orbit response significantly.

## 4. Machine learning: methodology

**Figure 5:**
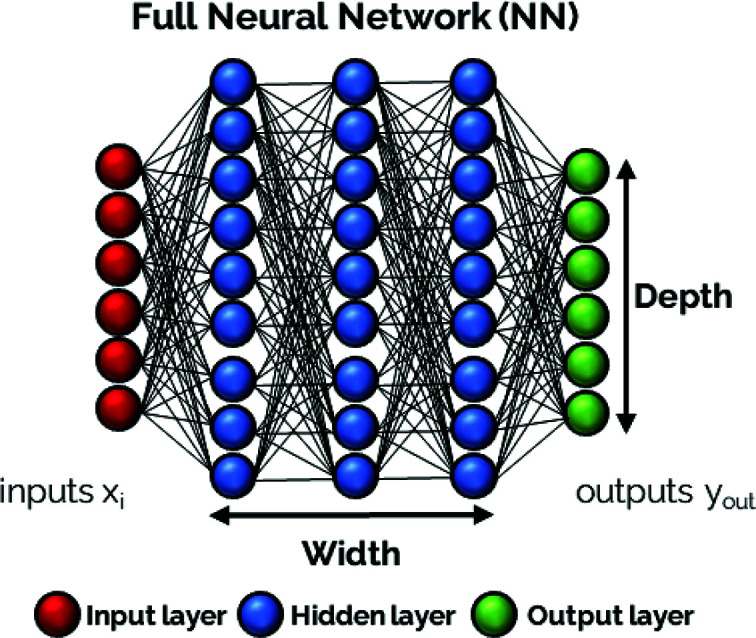
Schematic layout of a full NN. Each circle represents a neuron and a vertical string of neurons composes a layer. The parameters defining the architecture of the NN, depth (which may vary between the different layers) and width, are also shown.

Neural networks (NNs) , a special type of machine learning method (Mitchell, 1997 ▸), are powerful computational instruments that link input (*x*_*i*_) and output (*y*_*i*_) data. Many different NN architectures have evolved over recent decades (Alzubaidi *et al.*, 2021 ▸). In this work, we focus on densely connected feedforward NNs, where neurons are grouped in layers and each neuron from one layer is connected to all neurons in the subsequent layer. Therefore the number of layers (= width of the NN) and the number of neurons per layer (= height of a layer) are the first and most critical hyperparameters that need to be chosen. A schematic of such a neural network is depicted in Fig. 5 ▸.

A single neuron maps its inputs *x*_*i*_ to the output of this neuron *y*_*i*_ using the activation function *g*,
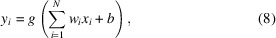
where the weights *w*_*i*_ and bias *b* are learnt through optimization, and *N* is the number of inputs. In this work, we use for *g* the ELU (exponential linear unit) activation function, defined as 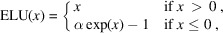
with α = 1 as the default value. The outputs of neurons in the previous layer serve as inputs to neurons in the next layer, forming the densely connected structure.

We followed a three-step approach, where the data are split into independent sets, Data_TRAIN_, Data_VAL_ and Data_TEST_, each containing inputs and outputs. Data_TRAIN_ is used to determine the optimal parameters to minimize the loss function. After each epoch (a group of gradient descent iterations), Data_VAL_ is employed to evaluate the model’s performance up to that point in the optimization process. Specifically, the loss for Data_VAL_ is computed to detect overfitting, which occurs when the network starts memorizing the training data points instead of learning the general relationship between input and output. In this scenario, the training loss decreases, while the validation loss remains constant or increases. Data_TEST_ is used for subsequent testing once the model training is completed, providing insights into the model’s generalizability. Considering the large variation in the data values, they were scaled from 0 to 1 using MinMax scaling.

The training of the NN is based on the minimization of a loss function whose definition depends on the specific problem considered. In our case we selected the mean squared error (MSE), defined as
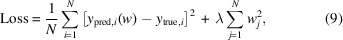
where *N* is the number of data points, *y*_pred,*i*_ and *y*_true,*i*_ are the output prediction and the training output values, respectively, *w* = (*w*_1_, …, *w*_*N*_) are the weights and λ is the regularization parameter. This last parameter is used to mitigate overfitting, since it limits the growth of the weights.

Beyond the regularization rate, many other parameters must be selected for optimal NN training. A crucial component of NN training is the selection of an appropriate optimizer algorithm. After some tests, we employed the *ADAM* optimizer (Kingma & Ba, 2015 ▸). The learning rate significantly influences the optimization process by determining the granularity of the updates, thereby affecting the computation speed. Typically, the optimization algorithm does not utilize all samples from the dataset simultaneously; instead, it processes data in subsets called batches at each iteration, making the batch size another critical hyperparameter. Additionally, the number of epochs is crucial for the optimization algorithm, and must be chosen either manually or through appropriate stopping criteria.

In the NN presented in this work we also implemented batch normalization and dropout layers between the dense connections. This reduces the convergence time of the training. Batch normalization (Ioffe & Szegedy, 2015 ▸) normalizes the data as they propagate through the network, preventing small changes in the weights from amplifying into significant variations in the output or the gradient. This normalization enables higher training rates, reduces the number of iterations needed and improves accuracy. Dropout layers (Srivastava *et al.*, 2014 ▸) are employed to reduce overfitting by randomly setting a fraction of the neurons’ outputs to 0 during the training. This strategy decreases reliance on specific neurons, thereby ensuring that all neurons contribute to the network output. After training, the dropout layers are deactivated, and all neurons are used for inference.

A possible limitation of the use of NNs is a change in the model after the training phase, which may make the use of the trained model impossible. In our case a typical example is the malfunctioning of one or more BPMs. We developed a way to overcome this issue, employing an autoencoder structure, described in Section 6.

To conclude, for all NN architectures the following hyperparameters need to be chosen: width and depth, activation function, loss function, regularization parameter, optimization algorithm, learning rate, batch size and number of epochs. We fine-tuned these parameters to achieve optimal performance, *i.e.* minimum loss and validation loss in the smallest possible number of epochs. We used *TensorFlow* (Abadi *et al.*, 2015 ▸) in conjunction with *Keras* (Chollet et al., 2015 ▸) and open-source Python libraries for the implementation of the framework.

## 5. Machine learning: application to orbit correction

We explored various scenarios with different machine perturbations and inputs. Specifically, this article focuses on two cases. In each scenario, the output corresponds to the corrector strength needed to steer the beam to the target orbit. The input can either solely consist of the beam orbit (type 1 network, N_1*a*_ and N_1*b*_) or include both the beam orbit and initial corrector strengths (type 2 network, N_2_).

### 5.1. Dataset generation

To generate the datasets, we perturbed the ideal lattice of the machine. We used both static perturbations, *i.e.* constant across all the data generation seeds, and dynamic perturbations, *i.e.* varying at each seed. Table 2 ▸ provides an overview of the type and magnitude of perturbations applied to generate datasets for the different networks.

We conducted simulations under conditions where machine elements exhibited both uncorrelated and correlated mis­alignments, and also perturbations occurring during synchrotron operation, like changes in tunes via variation of the quadrupole strengths and light wavelength changes via adjustment of the ID gaps. The orbit correction algorithms described in Section 3.1 assume prior correction of coupling. Conversely, the ML-based approach remains effective even in the presence of coupling within the machine. To demonstrate this capability, we intentionally introduced additional coupling into the lattice.

For each seed, we generated an orbit (as input for the NN) by introducing perturbations according to the distributions specified in Table 2 ▸. We then iteratively steered the beam towards the target orbit using the RM inversion approach until the maximum difference between the simulated orbit and the target one was less than 10 nm, unless otherwise specified. For each initial orbit, we recorded the corresponding corrector strengths as outputs. For the networks of the first type, we reset the corrector strengths to their initial values at each seed. Conversely, for the networks of the second type, we allowed the corrector strengths to vary freely without resetting them.

The first case corresponds to operation under a reference condition and making adjustments back and forth from it (for example changing the machine tunes and returning to the nominal set point). This corresponds to the networks of the first kind, N_1*a*_ and N_1*b*_. The second option corresponds to scenarios where we freely move around the operating condition. In this case, the output remains consistent with previous cases, but the input includes not only the BPM readings in both planes, as in the previous scenario, but also the initial strengths of correctors before the orbit correction. For SLS 2.0, this results in doubling the number of input values per data point. This corresponds to the network of the second kind, N_2_.

Our perturbations aimed to ensure large orbit excursions of the order of a few millimetres, where nonlinear dynamics is non-negligible, corresponding to corrector strengths of a few amperes across all scenarios considered for the data generation.

We generated the training, validation and test datasets. Data_TRAIN_ and Data_VAL_ were utilized for model development as detailed in Section 4. Subsequently, the trained model was applied to estimate the corrector strengths based on the orbits in Data_TEST_. In our ML model, the input consists of orbit data while the output corresponds to the corrector strengths. Under a standard testing approach, the evaluation would conclude at this stage. However, we extended the analysis by implementing the calculated corrections on the orbits in Data_TEST_ within the machine’s lattice. This approach offers two advantages: it enables direct evaluation of the orbit correction scheme’s performance on the beam by measuring orbit excursions rather than corrector strengths, and it facilitates the transition to future real-data implementation of the ML-based method, where BPM and corrector strength readings from the control system would replace those obtained by simulated data.

In the following subsections, we will focus on evaluating the performance of the networks belonging to the first kind, N_1*a*_ and N_1*b*_.

### 5.2. Neural network optimization and performance

We conducted a detailed study of network N_1*b*_, optimizing its hyperparameters and convergence speed. We prevented overfitting by applying techniques such as batch normalization and dropout layers, as discussed in Section 4. While we did not extend the optimization efforts for network N_1*a*_ to the same extent (especially in terms of convergence speed) as for the other considered cases, we used the results obtained from it to compare the ML performance with the pure RM-based method, explore its combined usage with it and assess the robustness of the approach proposed in this work.

#### 5.2.1. Network N_1*a*_

**Figure 6:**
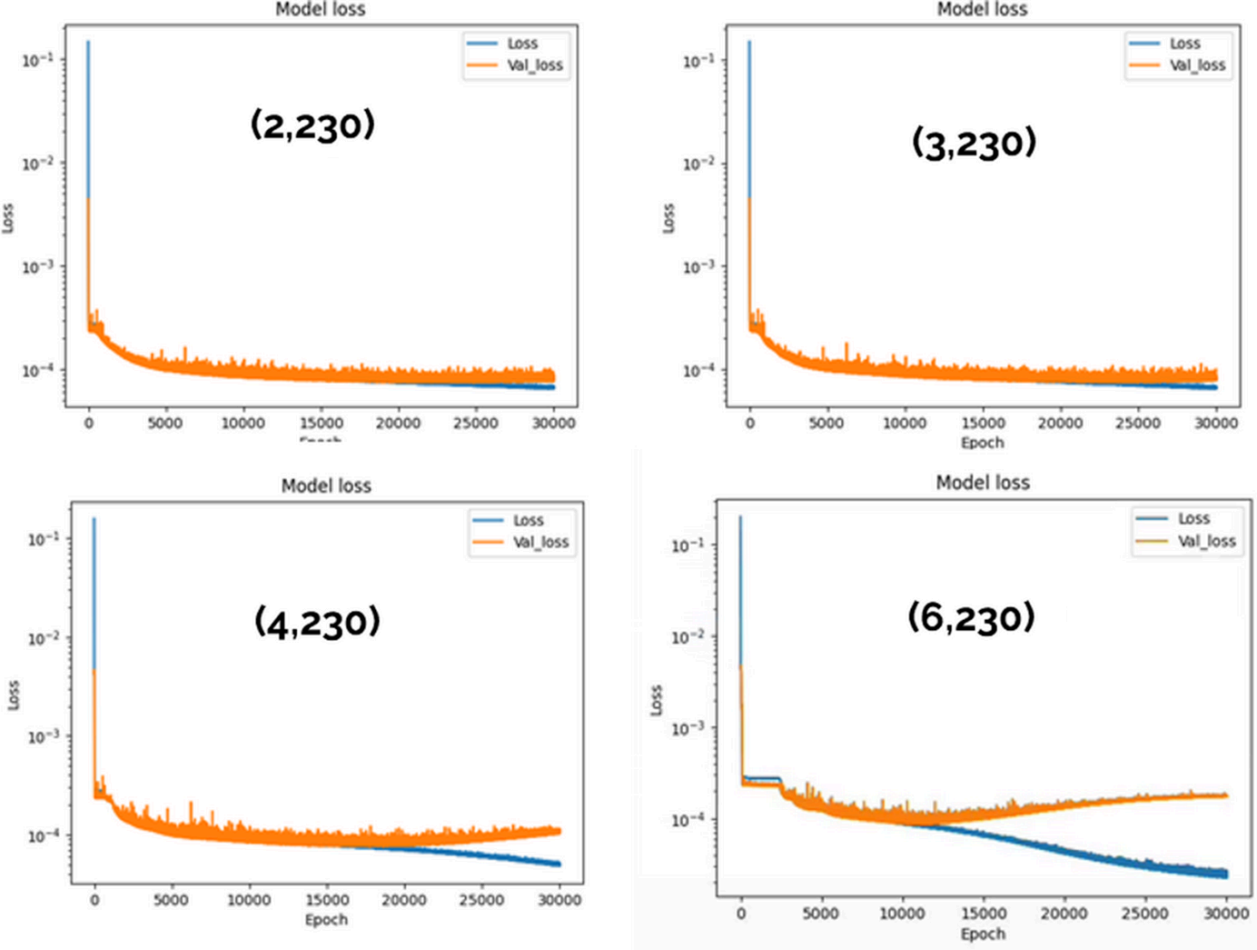
Loss and validation loss when varying the complexity of the network architecture (network N_1*a*_). The first and second numbers reported in parentheses in the plots indicate the width and the depth of the hidden layers, respectively.

We optimized network N_1*a*_ by performing a grid search over all relevant hyperparameters, selecting those minimizing the loss and also maintaining a small validation loss. During the training/validation phase, we observed that networks with insufficient complexity tended to underperform, resulting in high training and validation losses. Conversely, excessively complex networks exhibited overfitting, as showed by a significant increase in validation loss despite a continued decrease in training loss. With these considerations, we selected a network architecture that balanced performance (low losses) with simplicity, avoiding unnecessary complexity. Our primary criterion for choosing the architecture was achieving minimal loss while mitigating overfitting (small validation loss). This behavior is illustrated in Fig. 6 ▸, where overfitting appears in the most complex architectures. For instance, in the bottom right-hand graph, the validation loss begins to increase after approximately 10000 epochs, even as the training loss continues to decrease. Based on these considerations, we selected a network architecture with width and depth equal to 2 and 230, respectively, for all subsequent analyses.

**Figure 7:**
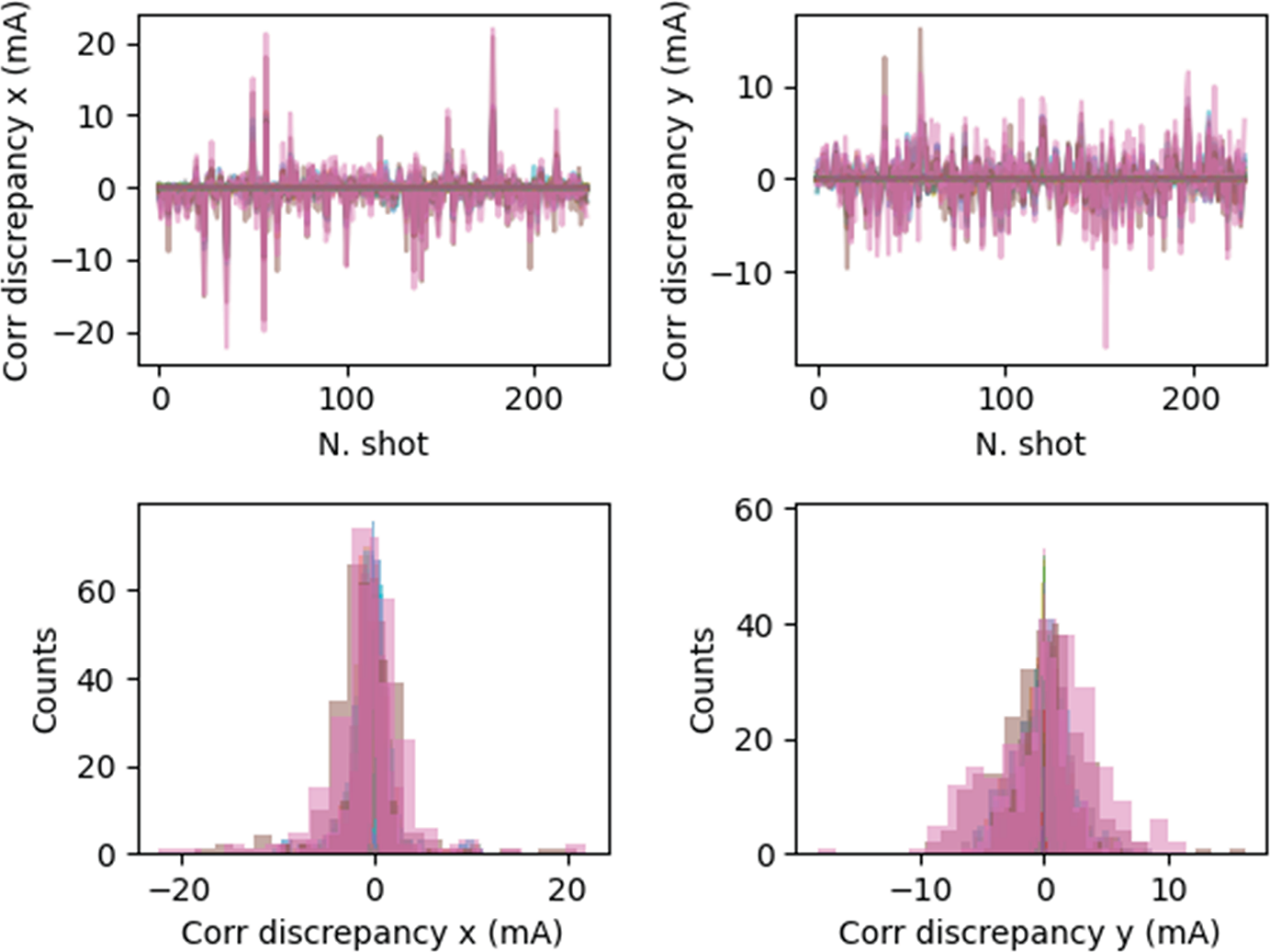
NN prediction for the network N_1*a*_: seed-to-seed (upper plots) and corresponding distributions (lower plots) of the trained model expectations with respect to the data output (Data_VAL_ and Data_TRAIN_ together) in both planes. Each color corresponds to a corrector.

We used both Data_TRAIN_ and Data_VAL_ to compare the expectations of the trained model with the outputs. In particular, Fig. 7 ▸ shows the peak-to-peak absolute difference between the model predictions and data not included in either the training or validation phases.

**Figure 8:**
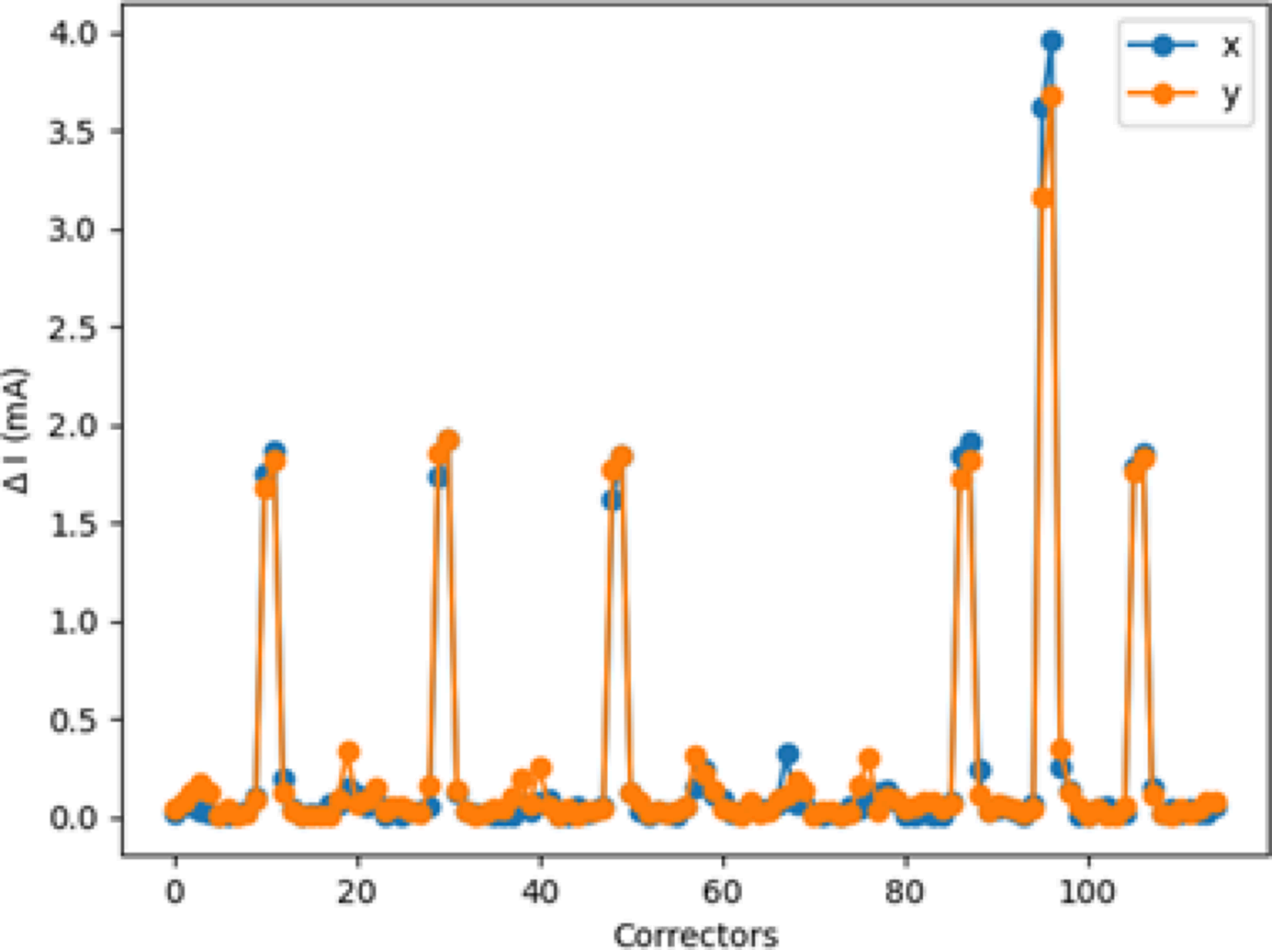
Network N_1*a*_. NN versus data comparison: standard deviation of the difference between the trained model expectations and the data output (Data_VAL_ and Data_TRAIN_ are used).

Fig. 8 ▸ shows the standard deviation of the absolute difference between the model prediction and the data as a function of corrector location along the ring. Even though the number of epochs required for convergence is probably not optimal, the performance of the trained model is deemed satisfactory for the purposes of our discussion, with the discrepancy between the model predictions and the actual data being of the order of one in a thousand at most at the ID locations.

**Figure 9:**
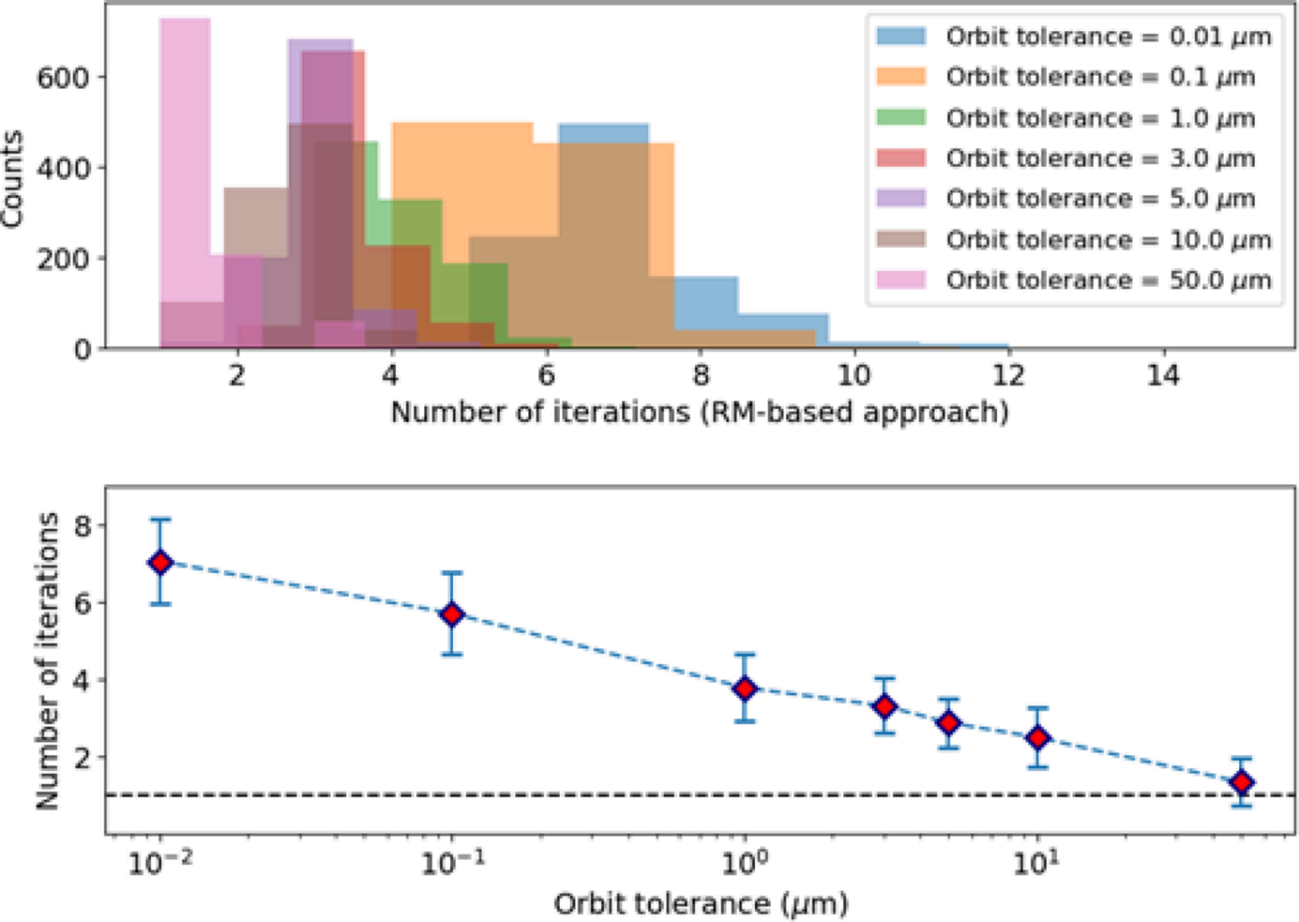
(Top) Distributions of the number of iterations necessary to correct the orbit, assuming different maximum orbit excursions in both transverse planes (orbit tolerances) in the presence of lattice perturbations introduced during generation of the dataset of the N_1*a*_ network. (Bottom) Mean (dots) and standard deviation (error bars) corresponding to these distributions.

**Figure 10:**
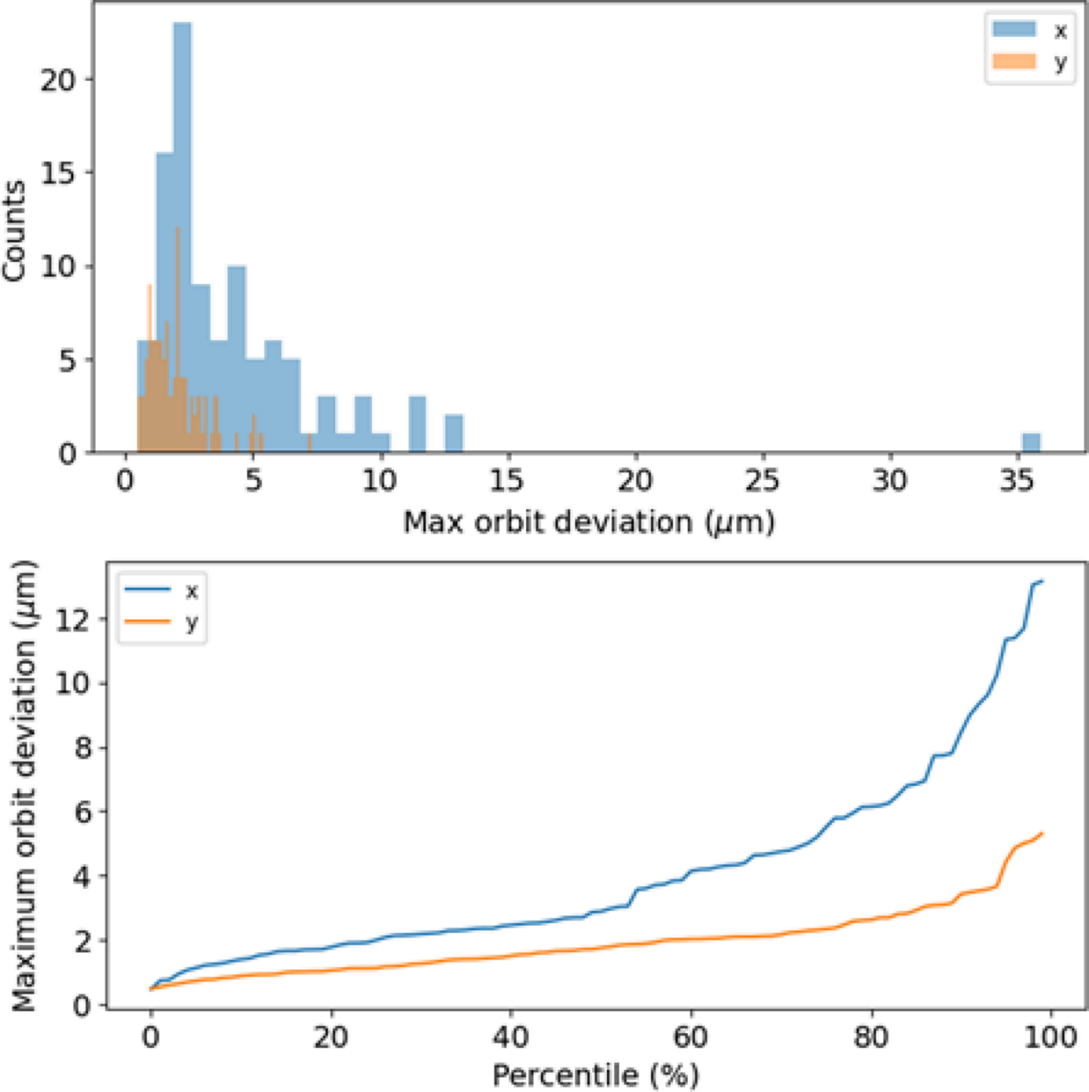
(Top) Distributions of the error on the corrected orbit using the trained model (network N_1*a*_) in both transverse planes. (Bottom) Maximum orbit deviation as a function of the percentile referring to these distributions. The majority of the seeds correspond to an orbit correction error below 5 m in both planes in a single ML iteration.

Fig. 9 ▸ shows the expected number of iterations required by a pure RM-based approach using matrix inversion to correct the orbit, assuming different tolerances on the maximum orbit excursion in both transverse planes and considering the same lattice perturbations introduced during the generation of the dataset for network N_1*a*_. On average between three and four iterations are necessary to correct the beam orbit to a maximum excursion within 1 µm to 10 µm, and up to seven iterations are required to achieve a correction of 10 nm. Fig. 10 ▸ shows that ML can steer the beam orbit down to a few micrometres in a single iteration.

**Figure 11:**
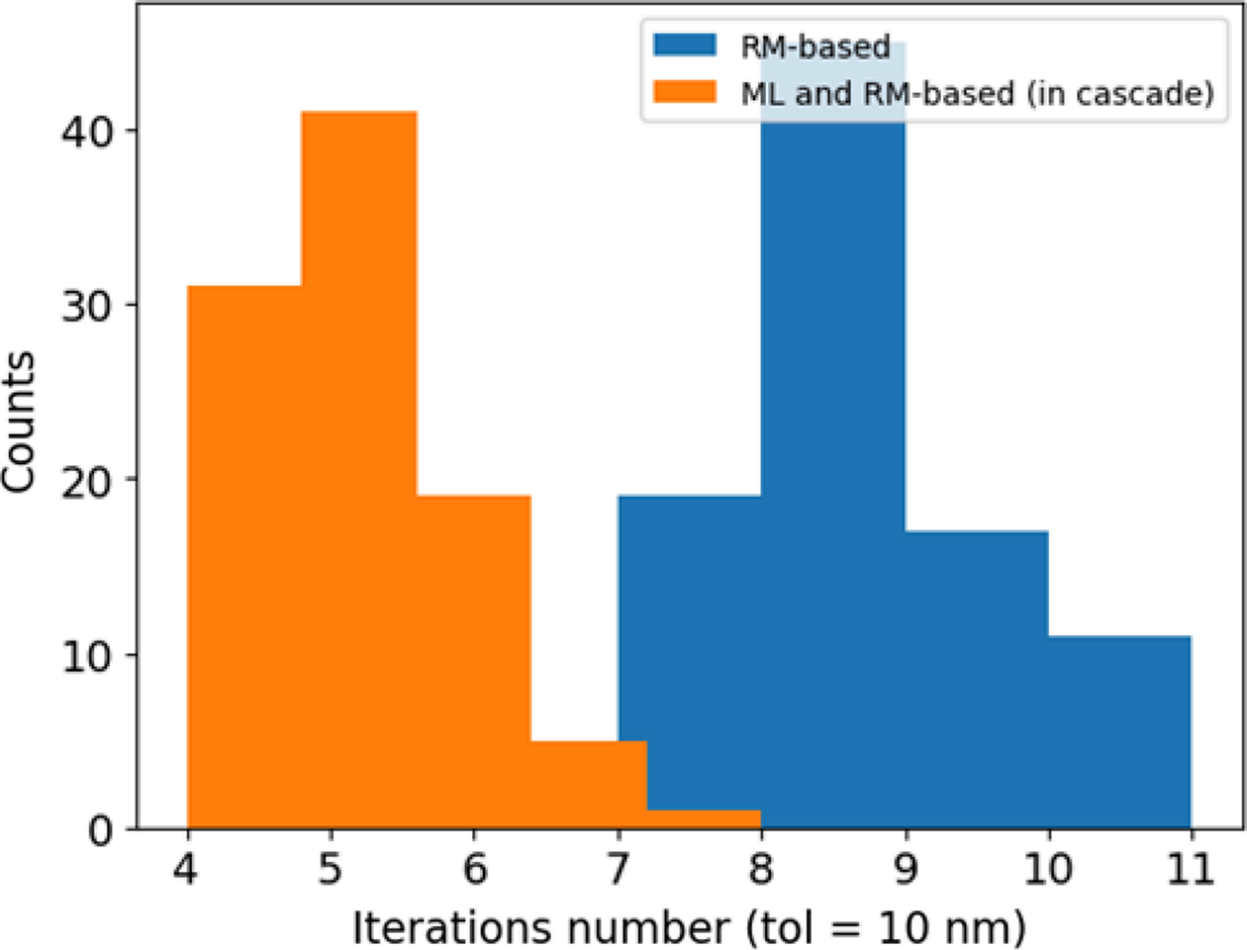
Comparison of the RM-based approach (blue) and the ML and RM-based method in cascade (network N_1*a*_, orange), showing the distribution of the number of iterations necessary to steer the beam assuming a maximum tolerance of 10 nm in both transverse planes.

ML may be utilized in various ways to correct the beam orbit: either as a standalone method, or as a preliminary phase followed by an RM-based or any other conventional approach applied in cascade. The latter option is particularly attractive because it exploits the ML approach’s capability to steer the beam efficiently close to the on-axis trajectory where the RM-based method becomes effective as well. The latter strategy allows an improvement in the machine’s stability to large perturbations, reducing the number of necessary iterations by a factor of two in our case, as shown in Fig. 11 ▸.

For all the studies described up to this point, we used the design RM, as this reflects the normal operating conditions of a running synchrotron during photon delivery. Measuring the RM typically requires half an hour or more at SLS, depending on factors such as the number of BPMs and correctors. However, relatively recent methods based on a sine-wave excitation of correctors at multiple frequencies have significantly expedited this process, reducing the time needed to measure a full RM to just a few minutes (Martin *et al.*, 2014 ▸; Yang *et al.*, 2017 ▸). We repeated the studies using the RM computed around the orbit at each seed (RM-seed) to verify that the improvement in the orbit correction speed was not due to the use of the design RM instead of the RM-seed.

**Figure 12:**
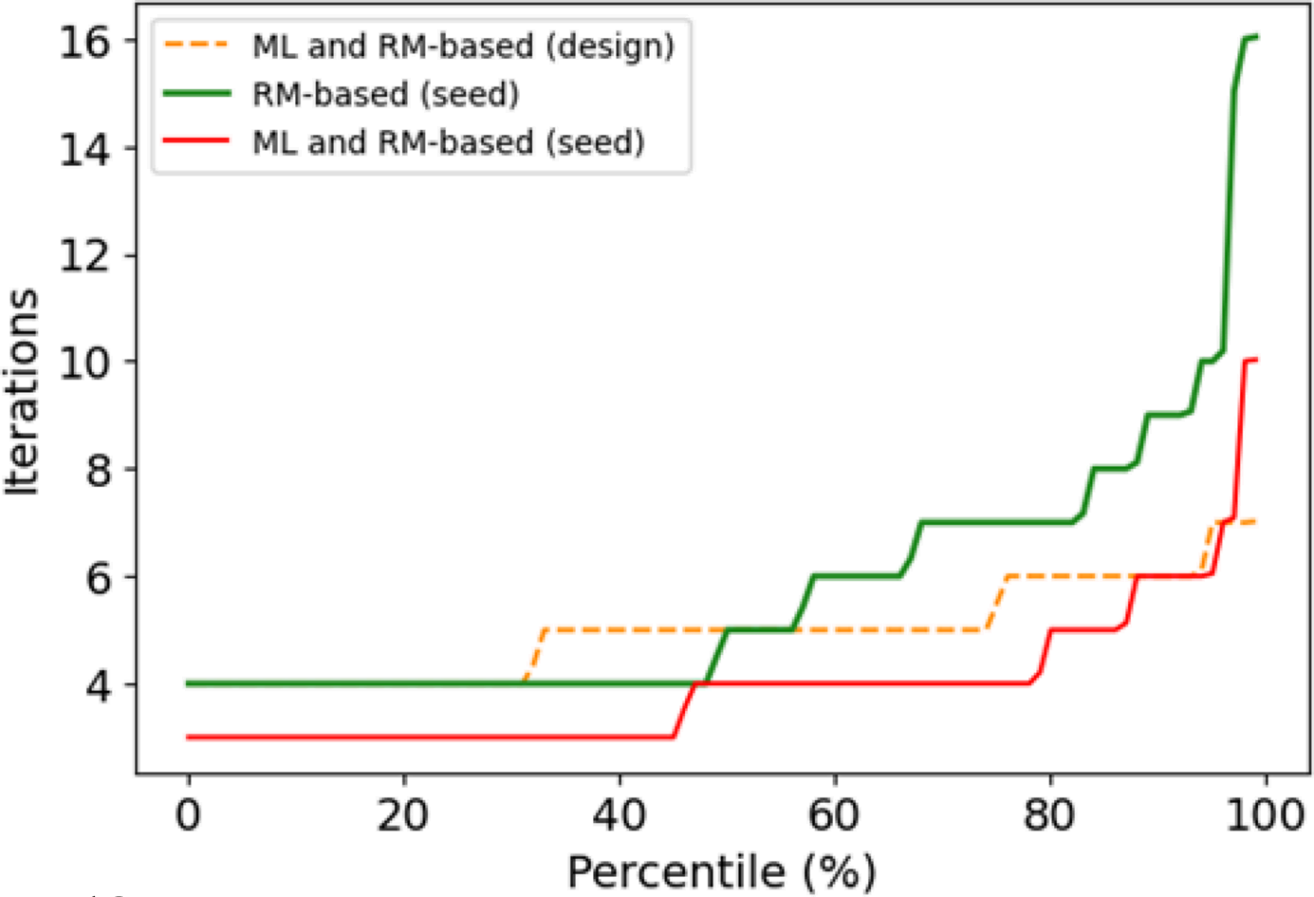
Number of iterations as a function of the percentile of the standalone RM-based approach, and the ML and RM-based approach used in cascade, assuming both the design and the seed RM (computed at each seed) for network N_1*a*_.

For the majority of seeds, employing the ML and RM-based methods in cascade proves to be advantageous compared to using the standalone RM-based approach, even when the RM calculated for each seed (RM-seed) is utilized, as shown in Fig. 12 ▸. This excludes the possibility that the reduction in the number of iterations required to steer the beam is due solely to the use of the design RM. Moreover, if the RM-seed instead of the design RM can be utilized for orbit correction (further reducing the time necessary to measure it), the acceleration achieved by the ML method can be even more significant.

#### 5.2.2. Network N_1*b*_

**Figure 13:**
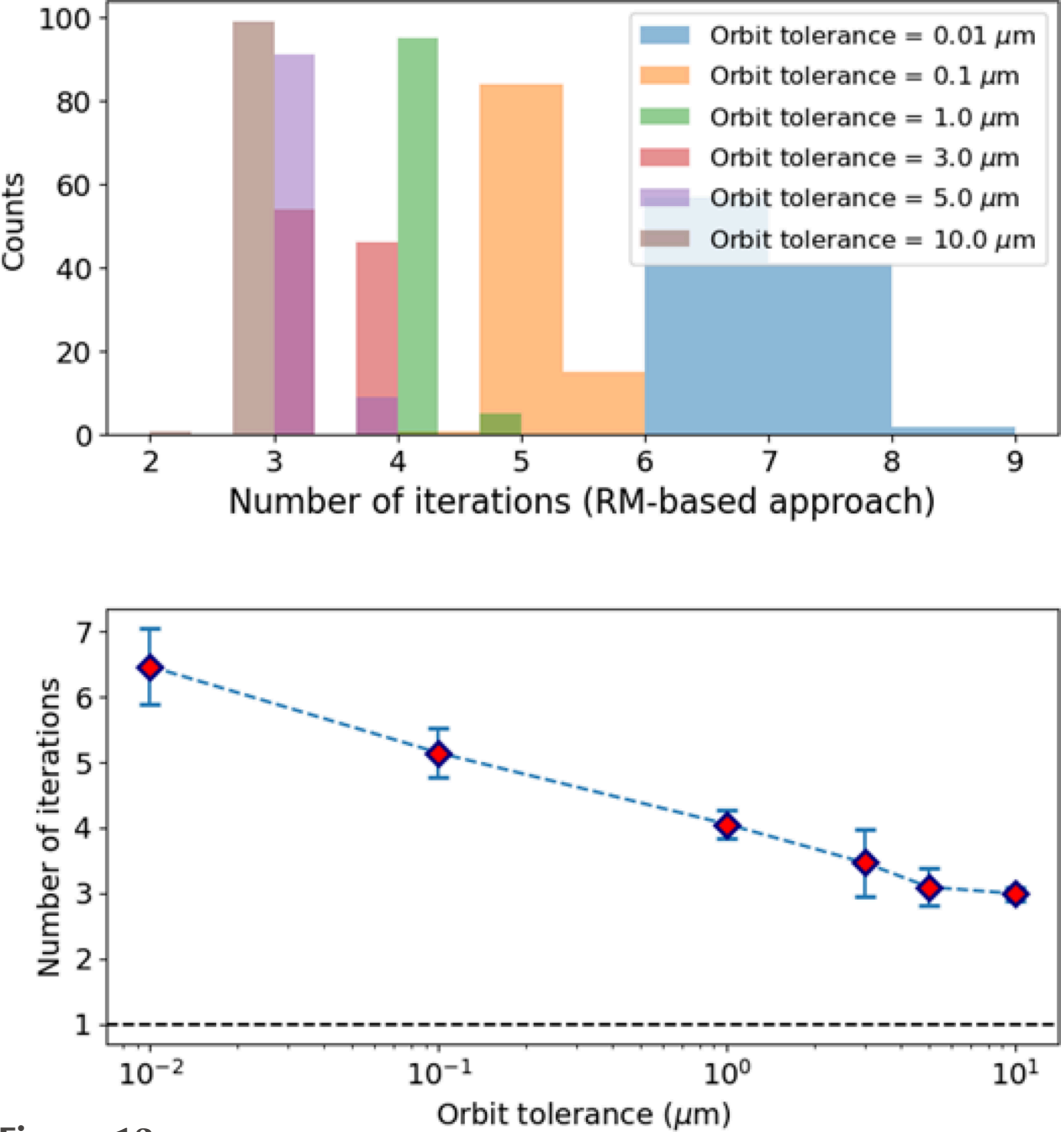
Usage of the trained NN to correct the orbit. (Top) Distribution of the number of iterations to correct the orbit assuming different values for the maximum orbit excursion. (Bottom) Mean and standard deviation of the distributions shown in the top plot.

We used different variables to generate the datasets for case N_1*b*_ to investigate different scenarios. We tuned the strengths of the variables to achieve similar beam orbit excursions and corresponding corrector strengths, but distributed differently along the ring. As a result, we obtained similar distributions for the number of iterations required to steer the beam using a fully RM-based approach based on RM matrix inversion, as evident from comparison of Fig. 13 ▸ and Fig. 9 ▸.

**Figure 14:**
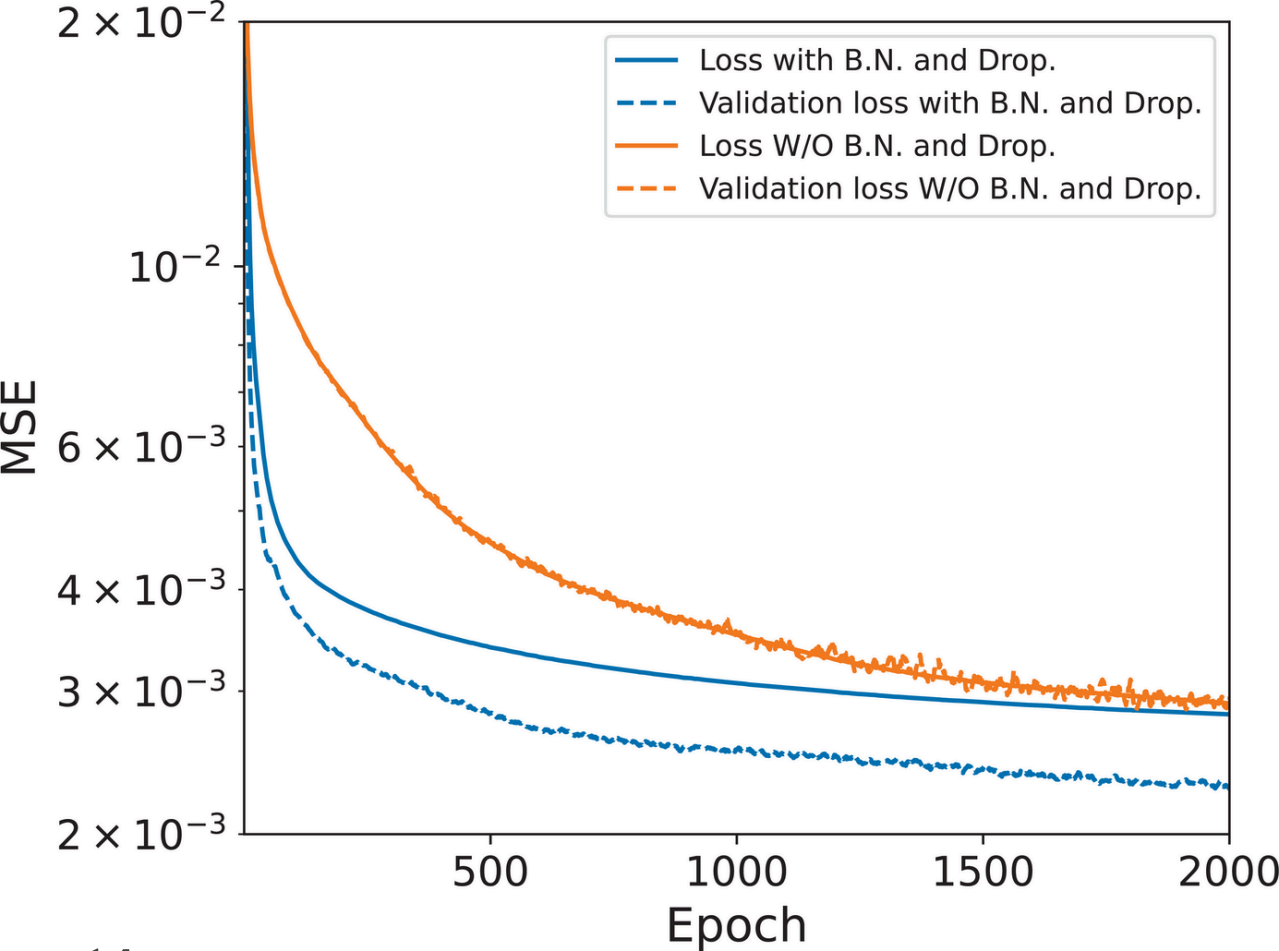
Loss and validation loss for network N_1*b*_ trained in the same way as N_1*a*_ and, in addition, with the implementation of batch normalization (B.N.) and dropout (Drop.).

Fig. 14 ▸ shows a comparison of a typical training plot between the case optimized using the same procedure as for network N_1*a*_ and that applying more advanced techniques such as dropout and batch normalization to improve the convergence of the training, as described in Section 4. The application of these techniques improves the convergence speed in terms of loss, and gives an even smaller validation loss than the standard optimization. Table 3 ▸ reports the optimized hyperparameters referring to the results reported in Fig. 14 ▸.

In the following we report the analyses obtained using the NN corresponding to the worst convergence speed, but they are also valid for the other case, which corresponds to an even better final validation loss.

**Figure 15:**
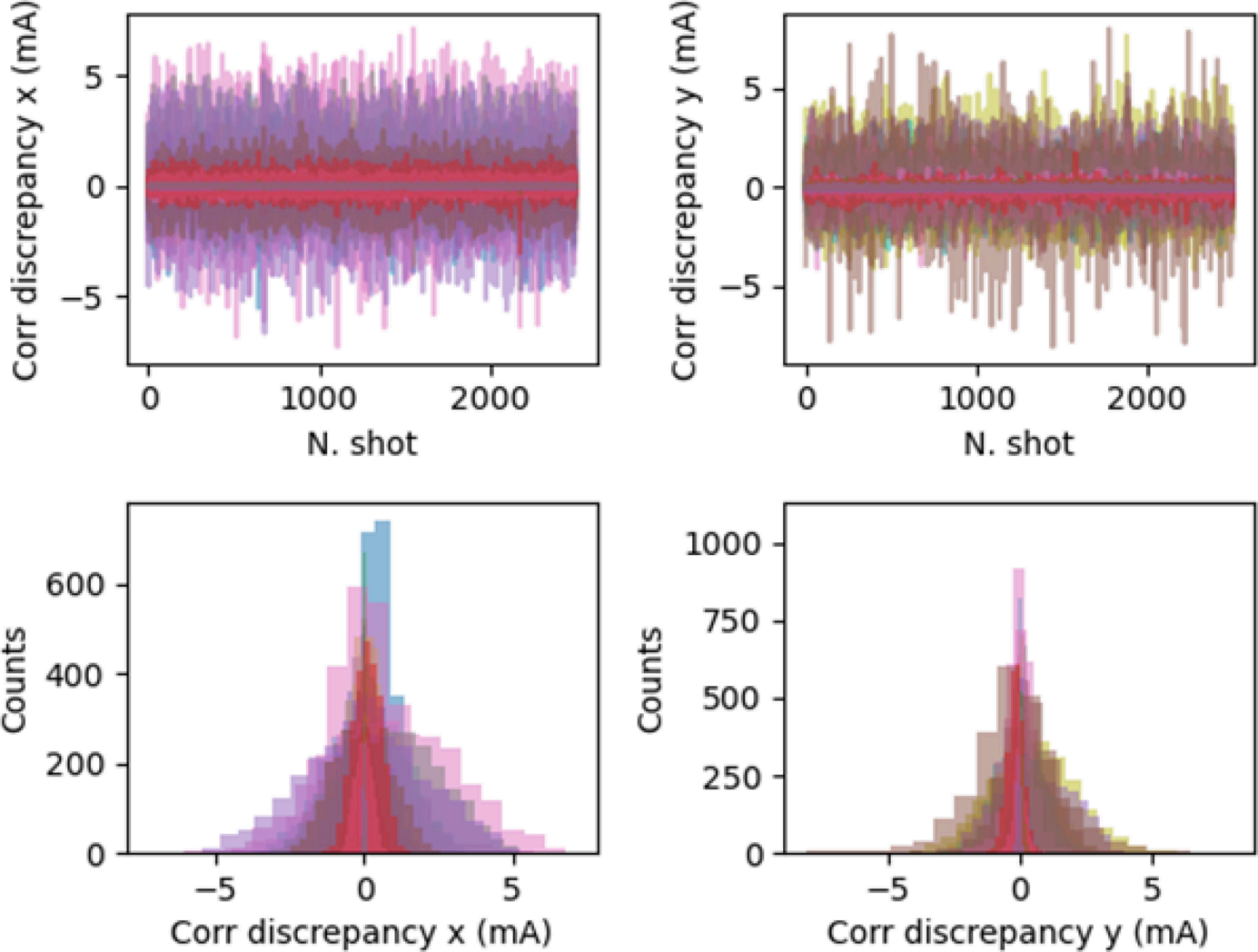
Comparison of NN versus data (network N_1*b*_). (Top) Seed-to-seed and (bottom) corresponding distributions of the model expectations with respect to the data output (Data_VAL_ and Data_TRAIN_) in both planes.

**Figure 16:**
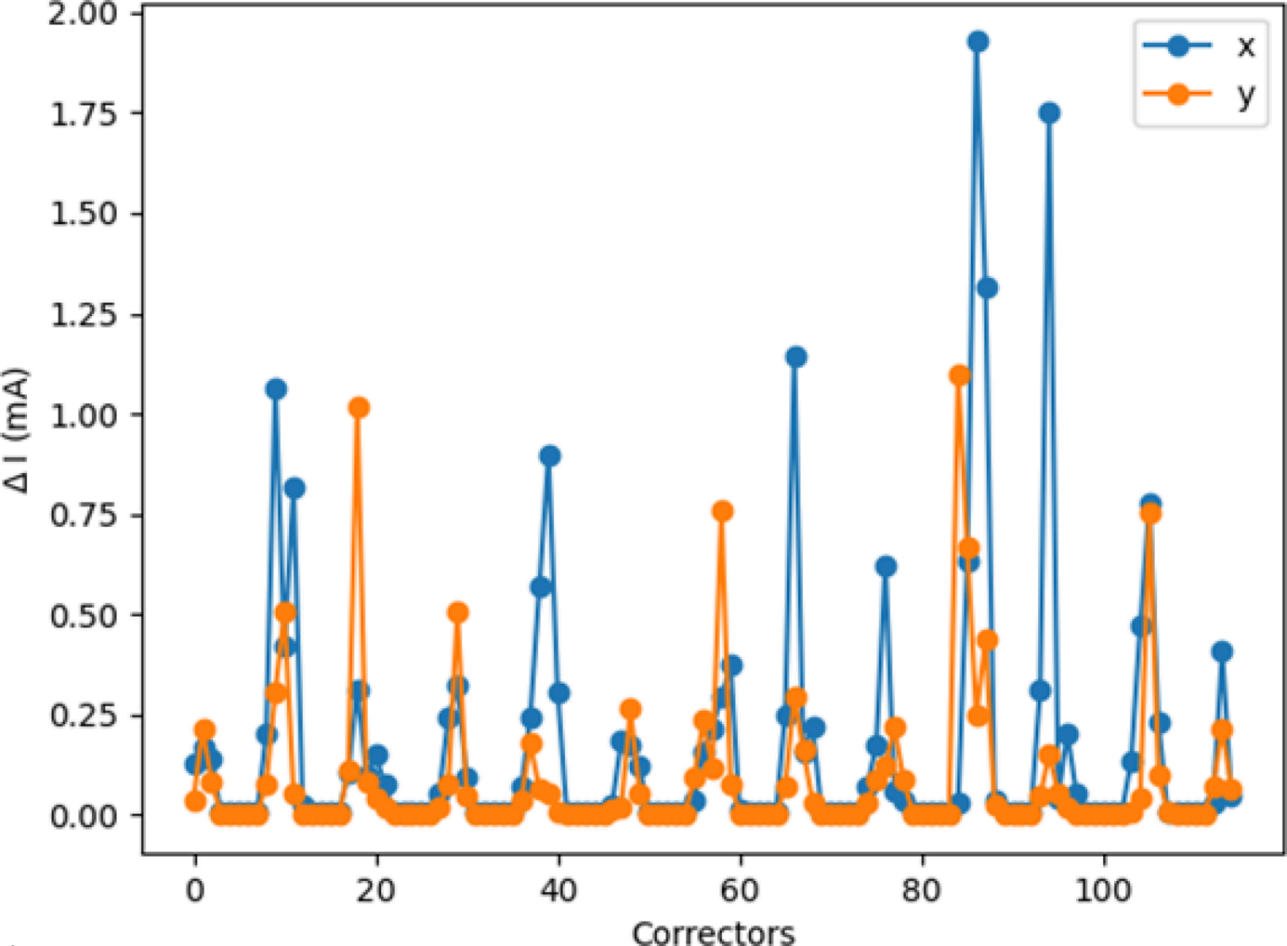
Network N_1*b*_. NN versus data comparison: standard deviation of the difference between the trained model expectations and the data output (Data_VAL_ and Data_TRAIN_ are used).

We obtained a very small (less than 5 mA) seed-to-seed discrepancy between the model predictions and the datasets for all cases considered, as shown in Fig. 15 ▸. This discrepancy corresponds to a standard deviation of about 2 mA in the worst case, and of the order of half this value for the majority of the shots, as reported in Fig. 16 ▸.

**Figure 17:**
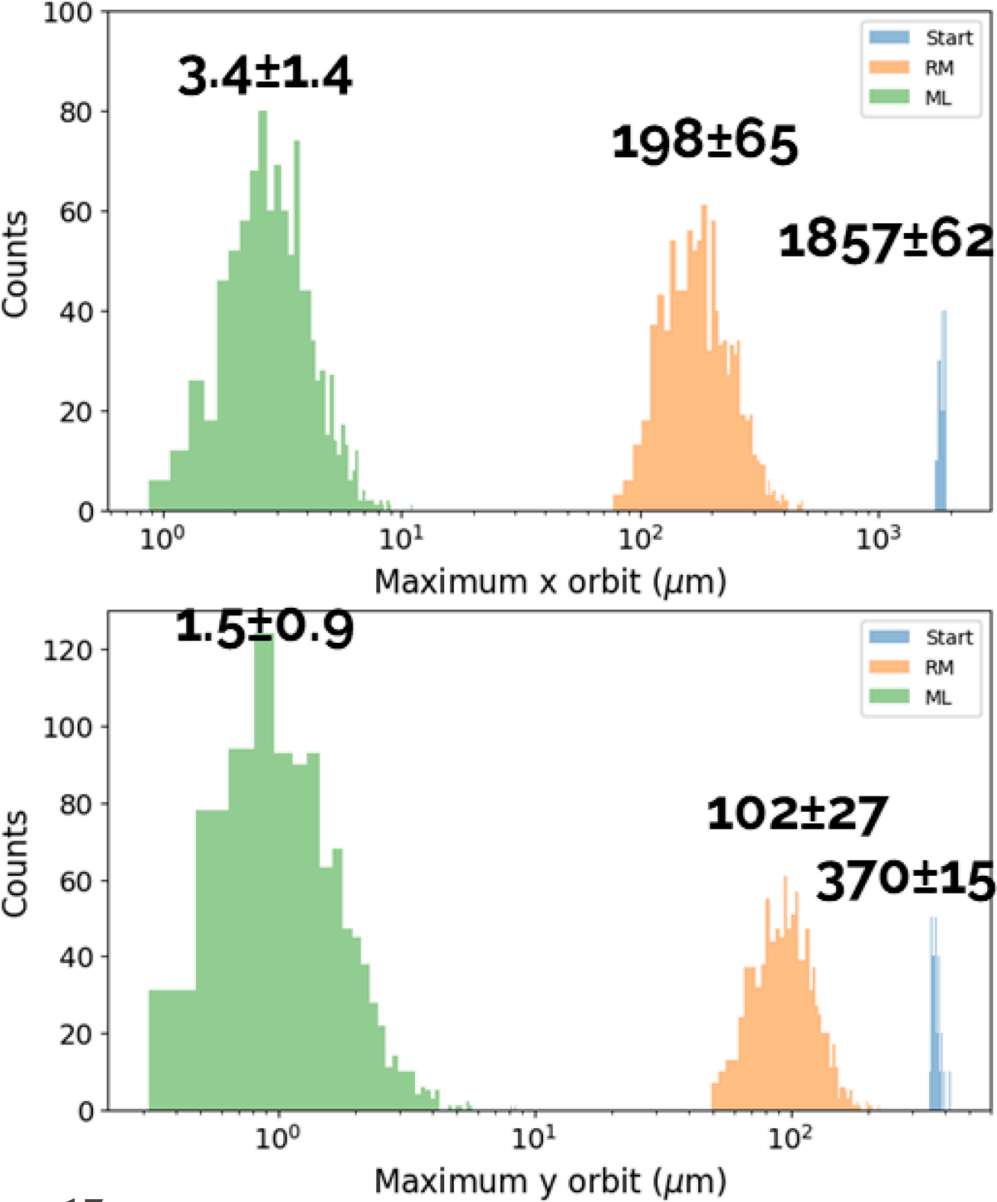
Comparison of the RM-based and ML methods (network N_1*b*_). Distributions of the maximum orbit deviation from the target orbit before any correction (blue), after one RM-based correction (orange) and after one ML correction (green). The upper and lower plots refer to the horizontal and vertical plane, respectively. The mean and standard deviation of each distribution are also reported in the plots.

We created several orbits by varying the parameters in the same manner as during dataset generation, and subsequently applied the trained model to steer the beam. Fig. 17 ▸ shows the results.

Starting from a maximum orbit excursion of the order of millimetres or a fraction thereof, the pure RM-based method brings the excursion down to a few hundred micrometres in a single iteration. The ML-trained model manages to steer the beam down to a few micrometres, corresponding to an improvement of about a factor of 70 in a single iteration. The RM-based approach would steer the beam orbit to the same values or even smaller, but it would require a larger number of iterations, between three and four according to Fig. 13 ▸. The inherent drawback of ML and NNs, in general, is that we cannot achieve a perfect model of the physics we aim to describe, but rather a model with some degree of error. This limitation is, to the best of our understanding, an undesirable yet unavoidable feature of any ML model. As a result, the most appealing approach to utilizing ML for beam steering is to employ it for the first iteration which, we have confirmed, sufficiently adjusts the beam off-axis to a degree where the nonlinear effects of the higher-order magnets typically present in synchrotrons are negligible. Therefore, we propose using ML solely for the first iteration and subsequently employing the more conventional and well established RM-based approach for further beam orbit correction. Combined use of ML (first iteration) and the RM-based approach (subsequent iterations) in cascade would allow a significant improvement in beam stability in cases of large orbit excursions.

#### 5.2.3. Extension to N_2_

So far, we have presented a scenario corresponding to the case of moving from the set point of the synchrotron and coming back to the same operating conditions (network of the first kind). We used this as our reference case: we verified the expected performance of the orbit steering using solely ML, exploiting it in combination with RM and computing the RM on the actual beam orbit, and optimized the network in terms of convergence. A synchrotron may also operate in a different way, characterized by changes without coming back to the initial set point. This case corresponds to the second kind of network. As mentioned above, this doubles the number of variables in the input, since the corrector strengths before the orbit correction must be included too. We verified that the increased complexity of the NN does not degrade the method’s performance in terms of the discrepancy between the trained model and the actual data, as shown by a comparison of Figs. 16 ▸ and 18 ▸.

We can therefore conclude that the performance of network N_2_ is equivalent to that of networks of the first kind. This further expands the applicability of the ML approach to correct the beam orbit.

## 6. Machine learning: model robustness to hardware failures

A critical challenge in applying ML to real-world systems is the potential change in data conditions over time, which can differ from the data used during training. If not properly addressed, this issue can render ML applications impractical. We examined the scenario where one or more BPMs malfunction after the data have been used for training. The NN architectures previously described cannot handle a different dimensionality and require retraining if a BPM value is missing. To address this, we modified our NN, making use of an autoencoder to enhance its robustness to such events.

**Figure 19:**
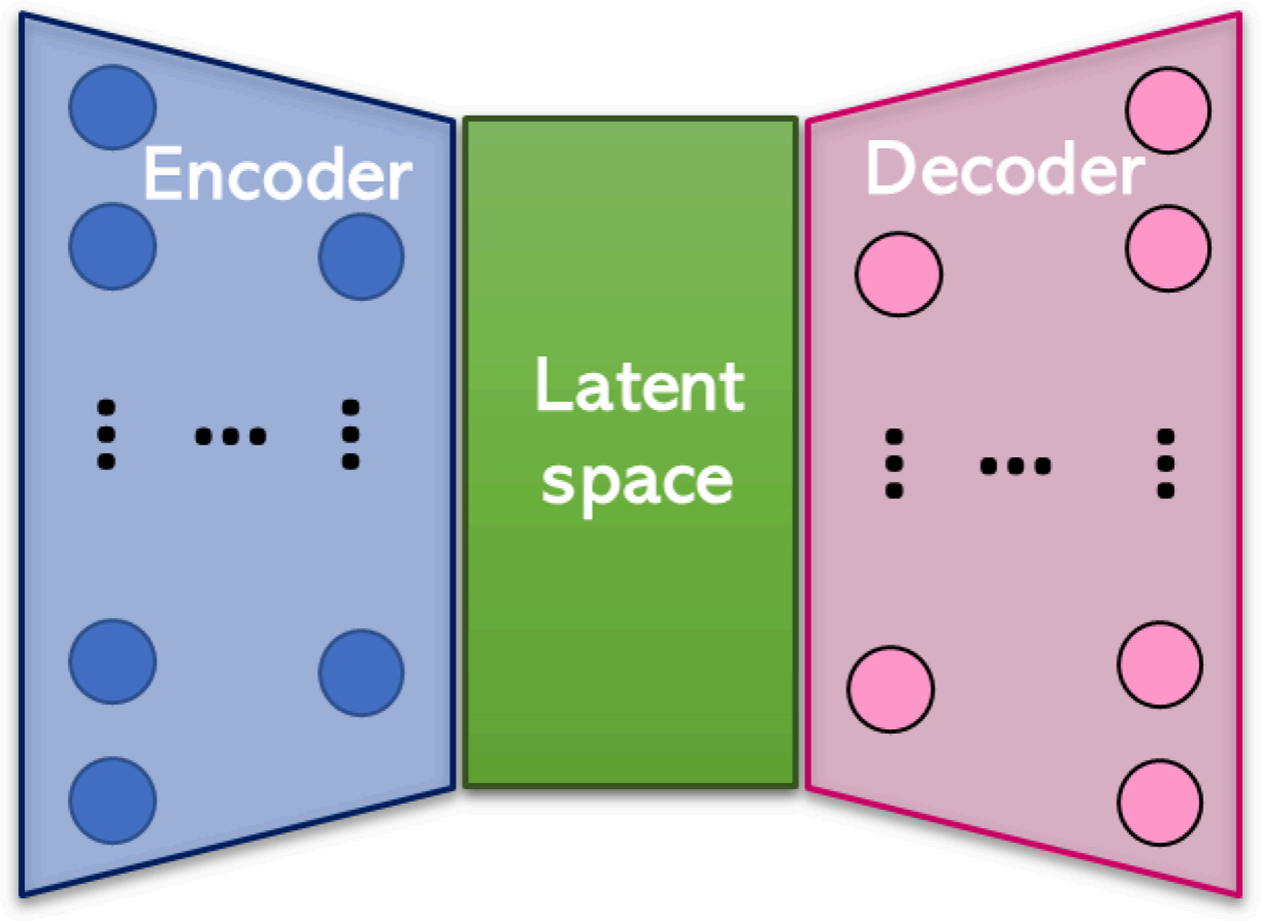
Schematic view of an autoencoder. The dimensionality of the NN varies along the different sections: the depth is reduced, constant and increased along the encoder, the latent space and the decoder, respectively. The circles represent the neurons.

An autoencoder is designed to encode input data into a lower-dimensional vector space, often referred to as the latent space, and subsequently to reconstruct the input from this encoded representation. This is achieved by decreasing the width of the dense layers during the encoding phase and increasing it during the decoding phase, as shown schematically in Fig. 19 ▸.

Ideally, the latent space should possess just enough dimensions to encapsulate all the essential information of the input vector. Our objective is to train the autoencoder such that the encoder can accurately represent the state of the beam in the latent space, even if some BPMs are broken. We can then utilize a new network to calculate the corrector strengths directly from the latent space, bypassing the need for the decoder. Instead of training the networks separately, we train both of them together as a single integrated network. This combined architecture takes the BPM measurements as input and it gives as output both the corrector strengths and the reconstructed BPM values.

We applied this technique to the data of network N_1*b*_. We replaced a fraction of the BPM readings with constant value data points that were not present in the training data, to simulate a broken device. For each selected data point, we substituted the measurement from a randomly chosen BPM and axis (*x* or *y*) with this constant value. Both the BPM and the axis with the faulty reading were selected randomly, as elaborated in the following. Generalizing to a scenario where a malfunctioning BPM affects both horizontal and vertical measurements can be done relatively easily without changing the results described in the following.

We employed an autoencoder architecture to reconstruct the missing BPM readings. Similar to the other case, we used the MSE as the loss function, but adding to it the MSE between the reconstructed BPMs and the original BPM readings. The autoencoder trained in this way also learns how to reconstruct accurately the readings of the broken BPMs.

**Figure 20:**
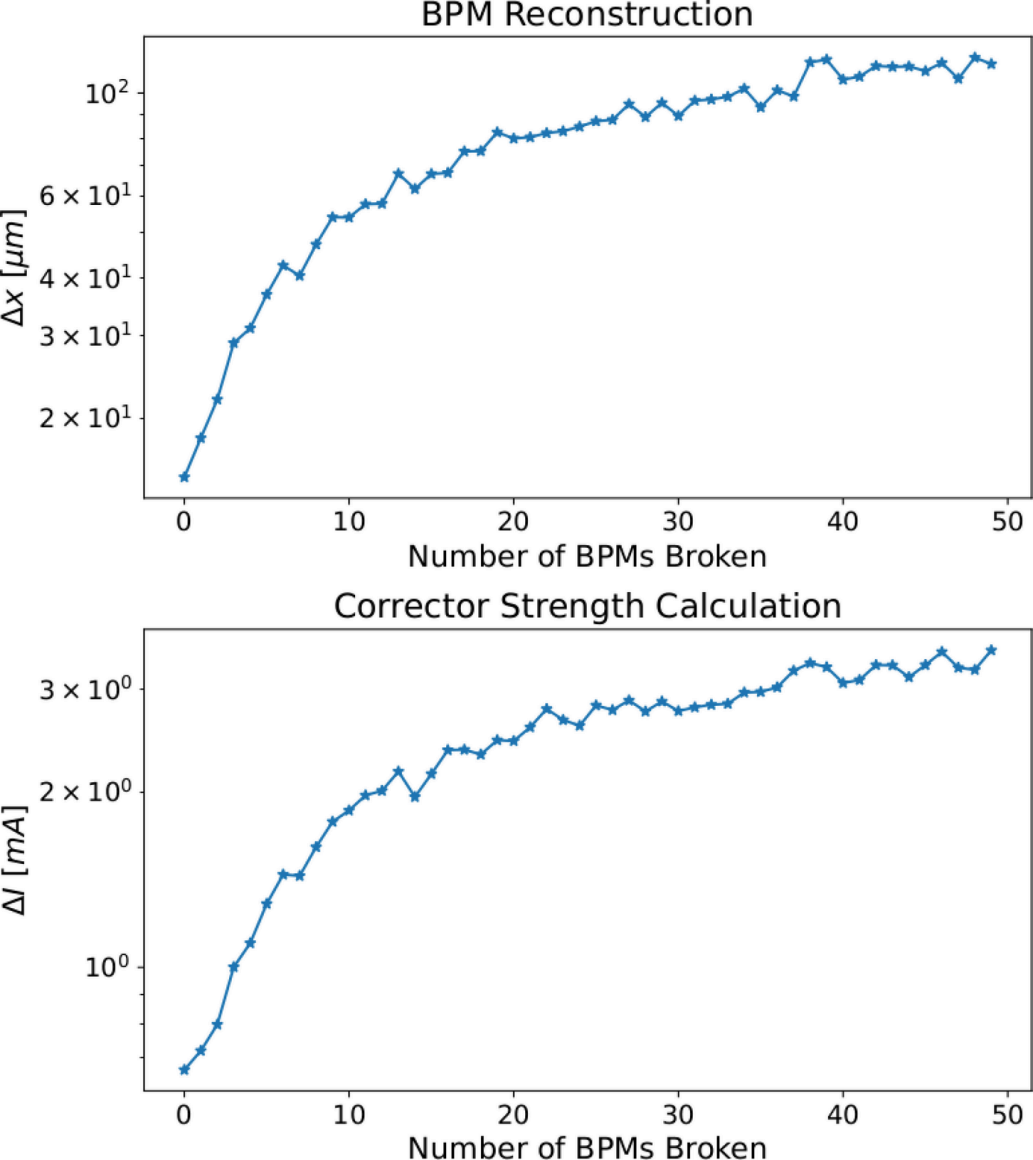
Mean error of the BPM reading reconstruction and corresponding corrector strengths as a function of the number of faulty BPM readings.

After careful optimization of the hyperparameters of the NN, we found that the best performing configuration is a symmetrical autoencoder with two layers in both the encoder and decoder, and a 30-dimensional latent space, *i.e.* a reduction of almost a factor of eight with respect to the previous networks. The additional section to calculate the corrector strengths consists of two layers as well. Fig. 20 ▸ shows the mean of the discrepancy between the computed corrector strengths and the generated data as a function of the number of BPMs giving a faulty reading, 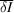, and the corresponding mean error on the BPM reading reconstruction, 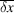.

Using the autoencoder NN, we obtained an error in the prediction of the corrector strengths of the order of a few milliamperes for up to three faulty BPM readings. This represents a step forward for the use of NN in real accelerators, because it makes us confident that we can use the NN even in cases of malfunctioning hardware, which may sometimes happen during the machine’s operation.

## 7. Conclusions

The RM-based approach is conventionally used for beam steering in accelerators. In machines equipped not only with linear but also higher-order magnets such as sextupoles and octupoles, the RM terms are influenced by the beam orbit through these higher-order magnets. As a result, the standard RM-based approach often requires multiple iterations to correct the beam orbit, in particular for large deviations.

Given the typically large number of variables involved, we explored the possibility of using an ML-based approach either as a standalone correction technique or in conjunction with RM-based methods. This approach is independent of the specific algorithm used for beam steering and uses past orbit corrections to adjust beam orbits efficiently. Moreover, it works well in the presence of machine element misalignments, coupling and other unknowns. We applied this approach to SLS 2.0. In the case of large perturbations (millimetre-scale excursions) the ML-based method predicts the corrector strengths needed to steer the beam with a precision of a few parts per thousand (maximum orbit excursions of the order of a few micrometres) in a single iteration step, corresponding to an approximately 70 times smaller orbit deviation compared with the prediction of the commonly used RM-based approach. Our investigations yielded consistent results across various types and strengths of perturbations. We also studied two operational scenarios – fixed set points and dynamic ones – and found comparable performances for the two cases.

Additionally, we enhanced the method’s robustness in the event of hardware failures, such as malfunctioning BPMs, which could potentially hinder ML applications. We developed strategies to mitigate these issues, further enhancing the attractiveness of the ML-based method.

The current ML approach shows very promising results for orbit correction. For future research, we plan to investigate different ML strategies as well. A combination of reinforcement learning with the simulation software used could enhance both efficiency and robustness. Additionally, ensemble methods that combine different ML models could offer further performance benefits. Exploring alternative NN architectures, such as convolutional-based networks, particularly those incorporating periodic boundary conditions, may provide a better representation of synchrotron dynamics. Finally, integrating uncertainty metrics and active learning could further optimize the model by refining predictions and adapting to evolving conditions.

Based on our findings, ML or, even better, ML integrated with an RM-based method, represents a robust tool for improving the SLS 2.0 stability in cases of large machine perturbations. Its applicability extends to other synchrotrons and machines featuring higher-order magnets beyond quadrupoles in their lattice.

## Figures and Tables

**Figure 18:**
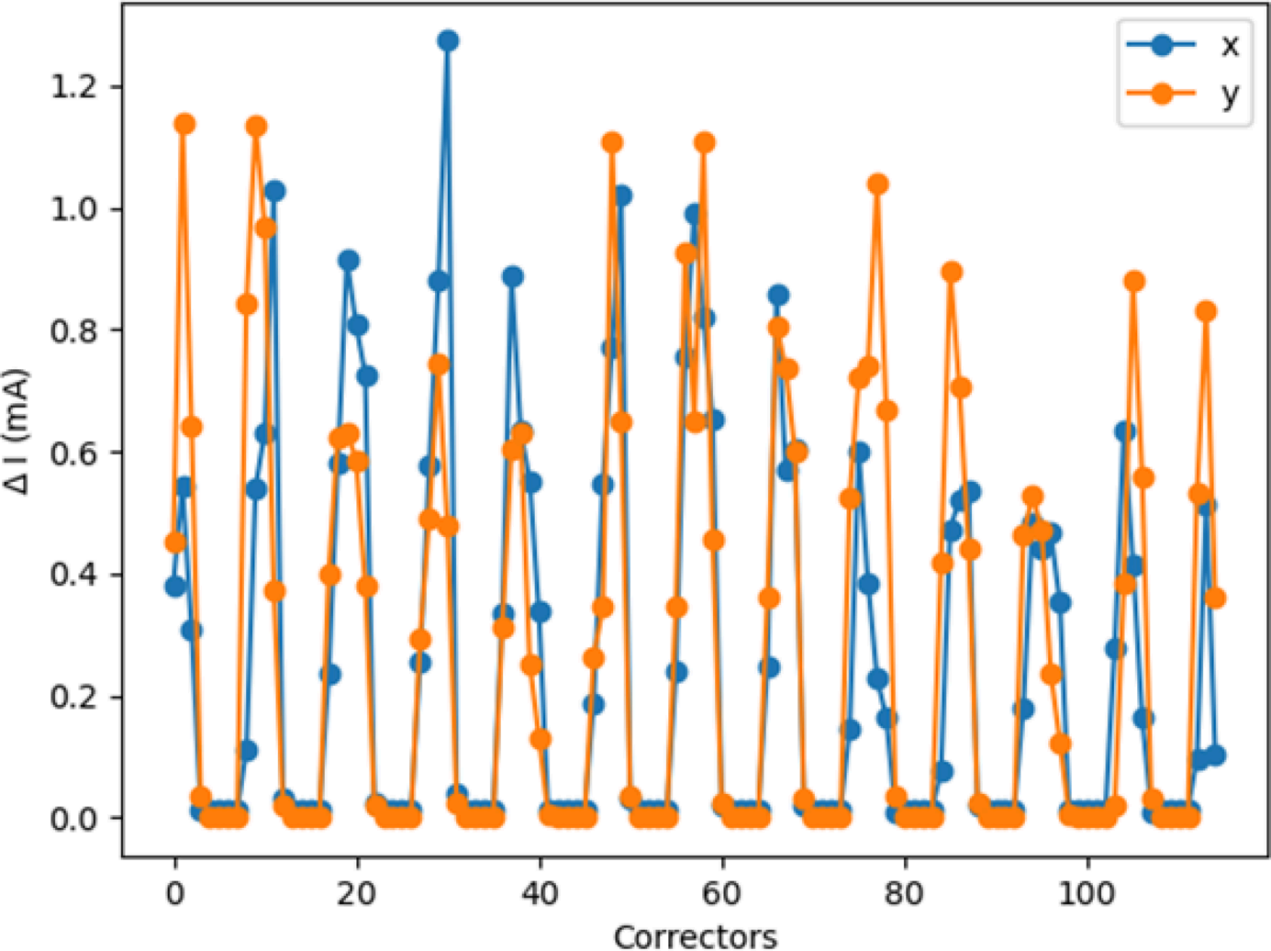
Network N_2_. NN versus data comparison: standard deviation of the difference between the trained model expectations and the data output (Data_VAL_ and Data_TRAIN_ are used).

**Table 1:** Comparison of the parameters of SLS and SLS 2.0 with emphasis on the most relevant for the discussion in this article H and V stand for horizontal and vertical, respectively.

	SLS	SLS 2.0
Energy (GeV)	2.4361	2.7000
Circumference (m)	288	288
Current (mA)	400	400
H, V emittances (pm)	5630, 10	135, 10
Operation mode	Top-up	Top-up
Number of H, V correctors	73, 73	115, 115
Number of H, V BPMs	73, 73	115, 115
Number of spare/test BPMs H, V	2, 2	20, 20
Corrector angle H, V (µrad A^−1^)	121, 96	120, 80
Maximum H, V kick strength (µrad)	850, 670	600, 400

**Table 2:** Perturbations introduced in the SLS 2.0 design lattice for the generation of the datasets The datasets are generated using the virtual accelerator (N_1*a*_) and *pyAT* (N_1*b*_ and N_2_). The type of perturbation, static (S) or dynamic (D), is also reported. The majority of the perturbations are defined as fractions of the machine imperfections used for the SLS 2.0 performance evaluation (Gaussian distributions truncated at two standard deviations) (Streun *et al.*, 2023 ▸). To them we added some extra coupling by varying the quadrupolar strength of the first sextupole from the injection, and extra kicks in both transverse planes to simulate residual dipoles introduced by the insertion devices (IDs) as functions of their operating gaps.

	N_1*a*_	N_1*b*_, N_2_	Type
Element-to-element (µm r.m.s.)	0.9	15	S
Girder center (µm r.m.s.)	1.8	30	S
Girder-to-girder (µm r.m.s.)	0.6	–	S
BPM offset (µm r.m.s.)	9	–	S
Magnet rotation (µrad)	9	150	S
BPM rotation (mrad r.m.s.)	0.3	–	S
Extra coupling: k_1_ at Sext_1_ (m^−1^)	–	0.1	S
Relative *k*_1_ (%)	±0.5	±1	D
ID kicks (H and V)	Variable	–	D

**Table 3:** Optimized hyperparameters and results referring to the N_1*b*_ network, obtained from a grid scan

Parameter	Value
Width	2
Depth	230
Batch size	512
Regularization parameter (*l*_2_)	1 × 10^−8^
Learning rate	5 × 10^−5^
Dropout function parameter	0.1
Final loss	3.4 × 10^−3^
Final validation loss	2.9 × 10^−3^
